# Post-marketing safety evaluation of paroxetine: a real-world pharmacovigilance analysis based on the FDA adverse event reporting system

**DOI:** 10.3389/fphar.2026.1744180

**Published:** 2026-04-14

**Authors:** Santosh Chokkakula, Qian Guo, Xiaoyue Zhang, Jawaher Bin Jumah, Bommireddy Naveen, Bing Yang

**Affiliations:** 1 Department of Microbiology, Chungbuk National University College of Medicine and Medical Research Institute, Cheongju, Republic of Korea; 2 Department of Rhinology, The First Affiliated Hospital of Zhengzhou University, Zhengzhou, China; 3 Oncology Department 4, The First Hospital of Hebei Medical University, Shijiazhuang, Hebei, China; 4 Nursing Administration & Education Department, College of Nursing, King Saud University, Riyadh, Saudi Arabia; 5 Department of Chemical and Biological Engineering, Gachon University, Seongnam-Si, Republic of Korea; 6 Department of Cell Biology, College of Basic Medical Sciences, Tianjin Medical University, Tianjin, China; 7 Department of Medical and Nursing Science, International College, Krirk University, Bangkok, Thailand

**Keywords:** disproportionality analysis, FAERS, major depressive disorder, paroxetine, pharmacovigilance

## Abstract

**Objective:**

Paroxetine, a selective serotonin reuptake inhibitor widely used in the management of major depressive disorders and other psychiatric disorders, remains insufficiently characterized regarding its adverse event spectrum. Thus, this study wanted to conduct a comprehensive evaluation of its post-marketing safety profile by analyzing reports from the U.S. Food and Drug Administration Adverse Event Reporting System (FAERS).

**Methods:**

Adverse event data related to paroxetine were extracted from the FAERS spanning the period from the first quarter of 2004 through the second quarter of 2025. Demographic distributions and reporter characteristics were analyzed to describe the exposed population. Signal detection was performed using four established disproportionality algorithms, including Reporting Odds Ratio (ROR), Proportional Reporting Ratio (PRR), Bayesian Confidence Propagation Neural Network (BCPNN), and Multi-item Gamma Poisson Shrinker (MGPS).

**Results:**

A total of 39,404 adverse event reports associated with paroxetine were included in the analysis. Female patients accounted for the majority of cases (57.41%), followed by males (32.84%). The most frequently reported preferred term (PT) was “drug withdrawal syndrome,” categorized under systemic diseases and administration site conditions, with 6,601 cases. This event demonstrated strong positive signals across all disproportionality metrics (ROR = 27.27, PRR = 26.31, χ^2^ = 148,641, IC = 4.61, EBGM = 23.47). The highest signal strengths were observed within the SOC of congenital, familial, and genetic disorders. Notably, the PT “carcinogenic effect on offspring,” although reported only three times, exhibited markedly elevated values (ROR = 952.54, PRR = 952.52, χ^2^ = 712.89, IC = 7.90, EBGM = 238.88), indicating an extremely disproportionate reporting frequency compared with baseline levels. Most adverse events (12.49%) appeared within the first 30 days of paroxetine therapy prescription.

**Conclusion:**

The pharmacovigilance assessment of paroxetine systematically identified significant safety signals. These findings provide important post-marketing evidence to strengthen drug safety surveillance and facilitate more informed, patient-centered therapeutic decisions in clinical settings.

## 1 Introduction

Major depressive disorder (MDD) is a prevalent and disabling psychiatric condition characterized by persistent depressive mood, anhedonia, and associated cognitive and behavioral impairments (American Psychiatric Association, 2022; Friedrich, 2017; Marwaha et al., 2023). In 2021, depressive disorders affected more than 330 million individuals worldwide, representing approximately 4% of the global population. They remain among the leading contributors to years lived with disability (YLDs) and disability-adjusted life years (DALYs) across multiple regions (Ferrari et al., 2024). Epidemiological evidence indicates that women are about 1.5 times more likely to develop depression than men, and perinatal depression constitutes a critical and globally recognized public-health concern (Woody et al., 2017; Van Niel and Payne, 2020). Despite substantial therapeutic progress, depressive disorders continue to contribute significantly to premature mortality and suicide, with more than 700,000 deaths reported annually, underscoring their persistent impact on global mental (World Health Organization, 2024; McGrath et al., 2020).

Pharmacotherapy remains a cornerstone in the treatment of MDD. Paroxetine, a potent selective serotonin reuptake inhibitor (SSRI) introduced in the late 1980s, continues to be extensively prescribed for both depressive and anxiety-related conditions. It has received regulatory approval for the management of major depressive disorder, generalized anxiety disorder, panic disorder, post-traumatic stress disorder, and obsessive-compulsive disorder (Shrestha et al., 2023; Foster and Goa, 1997; Heydorn, 1999). While paroxetine demonstrates well‐established therapeutic efficacy, its distinct pharmacodynamic characteristics, marked by high affinity and potent inhibition of the serotonin transporter with negligible noradrenergic effects, have been linked to adverse outcomes such as discontinuation symptoms, weight gain, and sexual dysfunction (Chiappini et al., 2022; Seifert, 2025).

Pharmacologically, paroxetine exerts its antidepressant effect by potently inhibiting serotonin reuptake at presynaptic terminals, thereby enhancing serotonergic neurotransmission within cortical and limbic circuits involved in mood and anxiety regulation (Stahl, 2000; Gunasekara et al., 1998). Nevertheless, real-world clinical findings indicate that optimal dosing parameters and long-term safety profiles may differ among patients due to interindividual variability in pharmacokinetics and pharmacodynamics. The substantial affinity of the drug for muscarinic cholinergic receptors, along with strong inhibition of the serotonin transporter, contributes to anticholinergic side effects, cognitive slowing, and sedation (Fujishiro et al., 2002; Cavanah et al., 2024). These observations emphasize the importance of large-scale post-marketing pharmacovigilance studies to characterize the real-world safety landscape of paroxetine beyond the constraints of controlled clinical trials.

Paroxetine labeling carries a boxed warning regarding the elevated risk of suicidal thoughts and behaviors in adolescents and young adults, consistent with meta-analytic findings demonstrating higher odds of suicide-related events among antidepressant users younger than 25 years (US Food and Drug Administration, 2004; Bauer et al., 2006). Discontinuation syndrome remains one of paroxetine’s most clinically notable adverse outcomes, typically presenting as dizziness, sensory abnormalities, insomnia, or irritability within a few days of dose reduction or withdrawal (Fava et al., 2015; Papoutsaki et al., 2021). Additional safety concerns include hyponatremia, serotonin syndrome, QT-interval prolongation, metabolic disturbances, and weight gain reported during clinical and post-marketing surveillance (Otsuka, 2017; Inoue et al., 2023). Overdose and toxicity cases have documented serious complications, such as seizures, cardiac arrhythmias, and profound central nervous system depression (Pütz et al., 2025; Florou et al., 2025).

Randomized controlled trials often lack statistical power and observational duration to identify rare, delayed, or context-dependent adverse drug reactions due to restricted sample sizes, controlled conditions, and selective inclusion criteria. Consequently, longitudinal real-world pharmacovigilance resources such as FAERS serve as critical tools for detecting unrecognized or low-frequency safety issues across heterogeneous patient populations. The present study uses FAERS data from 2004 to 2025 to systematically assess the post-marketing safety profile of paroxetine. Disproportionality analysis methods were applied to quantify statistical associations between paroxetine and reported adverse events relative to background reporting rates across the entire FAERS dataset. Elevated disproportionality indices indicate drug–event combinations reported more frequently than expected, suggesting potential safety signals warranting further evaluation. This comprehensive approach enables the identification of both established and emerging paroxetine-related risks, supporting evidence-based pharmacovigilance and informing safer therapeutic decision-making in clinical practice.

## 2 Materials and methods

### 2.1 Data source and study design

This retrospective pharmacovigilance analysis utilized adverse event data extracted from the FAERS database. FAERS is an open-access, spontaneous reporting system that compiles post-marketing safety information submitted by healthcare professionals, consumers, and drug manufacturers. Reports covering the period from the first quarter of 2004 to the second quarter of 2025 were retrieved in ASCII format from the FDA repository. The study specifically examined paroxetine, identifying cases through the generic names “Paroxetine”, “Paroxetine HCl”, “Paroxetine Hydrochloride”, “Paroxetine Mesylate”, and the brand names “Paxil”, “Paxil CR”, “Pexeva”, and “Brisdelle.” All adverse events were standardized using the Medical Dictionary for Regulatory Activities (MedDRA) Version 28.1, which offers a structured hierarchical classification from specific Preferred Terms (PTs) to broader System Organ Classes (SOCs).

### 2.2 Data preprocessing and quality control

**FIGURE 1:**
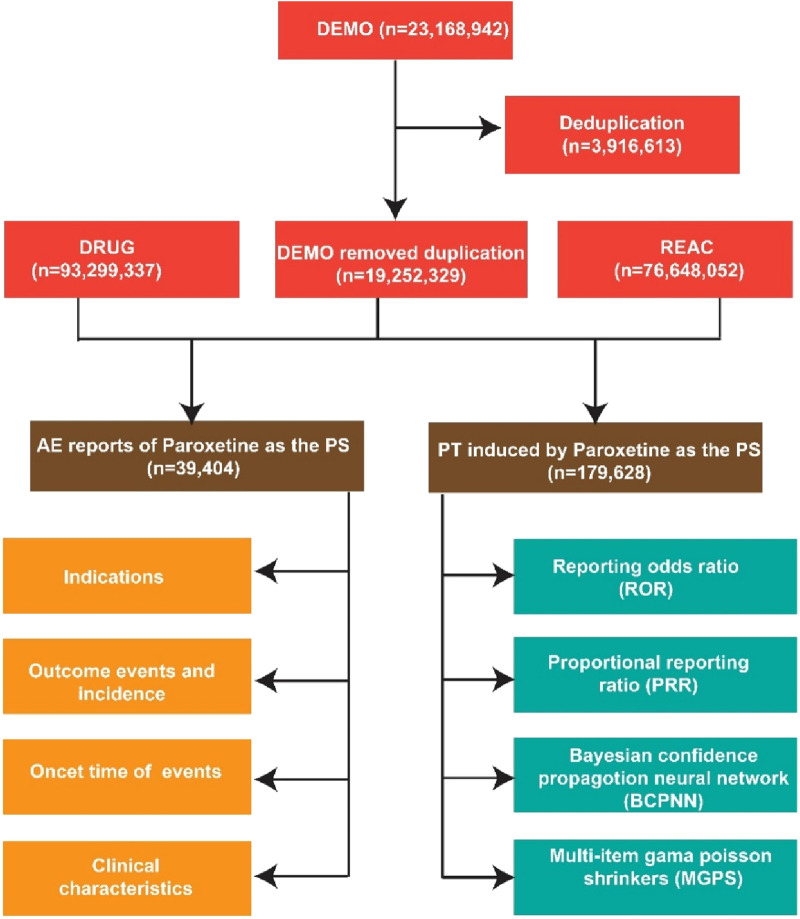
Flow diagram for the selection of AEs associated with PAROXETINE from the FAERS database.

Data cleaning was performed using SAS software version 9.4 to ensure the reliability of analyses. The FAERS database often contains duplicate case reports and inconsistent or missing data due to its voluntary reporting nature. Deduplication was conducted following FDA guidelines by sorting reports based on unique identifiers, including CASEID, PRIMARYID, and FDA_DT (report date). When multiple reports shared the same CASEID, only the latest report (determined by the highest FDA_DT) was retained. In cases where the CASEID and FDA_DT were identical, the submission with the highest PRIMARYID was kept, preserving the most current information. After deduplication, the cleaned demographic (DEMO), drug (DRUG), and adverse reaction (REAC) datasets were merged. Analysis was restricted to cases where paroxetine was declared the primary suspect drug to reduce confounding effects. Time-to-onset calculations were performed by subtracting the start date of paroxetine therapy from the adverse event occurrence date. Inconsistent cases, such as those with AE dates preceding drug start or missing dates, were excluded from analysis. Data from the first quarter (Q1) of 2004 to the second quarter (Q2) of 2025 were extracted from the FDA website in ASCII format. After deduplication and removal of deleted cases in accordance with FDA guidelines, a total of 19,252,329 DEMO reports and 39,404 reports for paroxetine (with 179,628 adverse events) were obtained. The data processing workflow is shown in Figure 1.

### 2.3 Disproportionality signal detection methods

To identify potential associations between paroxetine and reported adverse events, disproportionality analysis, a fundamental method in pharmacovigilance, was applied using four complementary algorithms, including Reporting Odds Ratio (ROR), Proportional Reporting Ratio (PRR), Bayesian Confidence Propagation Neural Network (BCPNN), and Multi-Item Gamma Poisson Shrinker (MGPS). The two-by-two contingency framework illustrating these calculations is summarized in Supplementary Table S1. These analytical methods assess whether a specific drug–event combination is reported more frequently than expected relative to the entire FAERS database, thereby indicating possible safety signals. Frequentist approaches (ROR and PRR) offer higher sensitivity in detecting frequently observed events, whereas Bayesian models (BCPNN and MGPS) better account for sparse data and minimize false-positive associations. Integrating these complementary algorithms strengthens the robustness and validity of signal detection outcomes.

### 2.4 Signal criteria and statistical analysis

Signals were defined according to internationally recognized thresholds, as detailed in Supplementary Table S2. For ROR and PRR analyses, a signal required at least three case reports, with the lower limit of the 95% confidence interval exceeding 1. For PRR, an additional criterion of a minimum ratio value of 2 was applied to designate a signal as clinically meaningful. The BCPNN method identified signals when the lower bound of the Information Component’s 95% credibility interval (IC025) was greater than zero, following World Health Organization recommendations. The MGPS algorithm considered a signal when the lower limit of the Empirical Bayes Geometric Mean 95% confidence interval (EBGM05) exceeded 2, providing a conservative threshold for rare associations. All computations were conducted using SAS and SQL for high-throughput data handling and statistical assessment. This integrated approach maintained an optimal balance between sensitivity and specificity in detecting signals of paroxetine-associated adverse events.

#### 2.4.1 Subgroup analysis of age, gender, and pregnancy- and fetal-related events analysis

To explore heterogeneity in adverse event (AE) profiles among individuals receiving paroxetine, stratified analyses were performed based on key demographic and clinical characteristics. Participants were grouped by age (<18 years, 18–60 years, and >60 years), sex (male or female), and geographic region to compare AE patterns across distinct populations. This approach enabled the detection of demographic-specific trends and the identification of subpopulations potentially at increased risk following paroxetine exposure. To provide a more granular assessment of pregnancy- and fetal-related events, a PT-level disproportionality analysis was also conducted.

#### 2.4.2 VigiBase data source and study design

In addition to the FAERS dataset, data was extracted from VigiBase, the World Health Organization’s global database of individual case safety reports, maintained by the Uppsala Monitoring Centre. VigiBase compiles spontaneous adverse drug reaction (ADR) reports submitted by national pharmacovigilance centers participating in the WHO Programme for International Drug Monitoring. Reports associated with paroxetine from 2004 to 2025 were retrieved to facilitate cross-database signal validation. Adverse events were coded according to the Medical Dictionary for Regulatory Activities (MedDRA, Version 28.1). All analyses were performed in accordance with WHO data-use regulations, using only aggregated and anonymized records.

#### 2.4.3 Comparative pharmacovigilance analysis

To evaluate the specificity of the AEs, signals detected for paroxetine and to mitigate potential reporting biases, a comparative analysis was conducted using sertraline as a reference drug. Sertraline was selected because, like paroxetine, it is a widely prescribed SSRI with overlapping clinical indications but possesses a distinct safety profile and a longer half-life. We compared the disproportionality signals of paroxetine against sertraline.

## 3 Results

### 3.1 Demographic and clinical characteristics of adverse event reports

After deduplication of the FAERS dataset from the first quarter of 2004 through the second quarter of 2025, a total of 39,404 AE reports associated with paroxetine were identified, encompassing 179,628 individual AEs. The principal characteristics of these reports are presented in Table 1. Females comprised the majority of cases (57.41%), while males accounted for 32.84%. Among reports with demographic data, the highest proportion of AEs occurred in adults aged 18–44 years (19.55%), followed by those aged 45–64 years (15.27%), individuals aged 65 years or older (11.36%), and those younger than 18 years (5.82%). The mean and median patient ages were 45.34 and 45 years, respectively, ranging from 0 to 104 years.

**TABLE 1:** Demographic and clinical characteristics of adverse event reports for paroxetine in the FAERS database (2004-2025).

Index	Number of cases (%)
Gender	
Female ((%)	22,623 (57.41)
Male (%)	12,941 (32.84)
Not specified (%)	3840 (9.75)
Age	
<18 (%)	2293 (5.82)
18-44 (%)	7,705 (19.55)
45-64 (%)	6,017 (15.27)
≥65 (%)	4478 (11.36)
Not specified (%)	18,911 (47.99)
Age (quantitative)	
N (Missing)	20,493 (18, 911)
Mean (SD)	45.34 (23.15)
Median (Q1, Q3)	45.00 (30.00, 62.00)
Minmax	0.00, 104.00
Reporting year	
2004 (%)	5717 (14.51)
2005 (%)	4950 (12.56)
2006 (%)	3867 (9.81)
2007 (%)	2651 (6.73)
2008 (%)	2644 (6.71)
2009 (%)	1561 (3.96)
2010 (%)	1467 (3.72)
2011 (%)	1710 (4.34)
2012 (%)	1281 (3.25)
2013 (%)	946 (2.40)
2014 (%)	953 (2.42)
2015 (%)	1889 (4.79)
2016 (%)	1602 (4.07)
2017 (%)	886 (2.25)
2018 (%)	1148 (2.91)
2019 (%)	1547 (3.93)
2020 (%)	1267 (3.22)
2021 (%)	893 (2.27)
2022 (%)	691 (1.75)
2023 (%)	668 (1.70)
2024 (%)	681 (1.73)
2025 (%)	385 (0.98)
Rapporteur	
Consumer (%)	21,871 (55.50)
Lawyer (%)	3211 (8.15)
Not specified (%)	1290 (3.27)
Other health professionals (%)	3461 (8.78)
Pharmacist (%)	2991 (7.59)
Physician (%)	6,580 (16.70)
Report on the continent to which the country belongs	
North America (%)	17,558 (44.56)
Europe (%)	10,688 (27.12)
Not specified (%)	8378 (21.26)
Asia (%)	2512 (6.37)
Oceania (%)	154 (0.39)
South America (%)	82 (0.21)
Africa (%)	32 (0.08)
Reporting countries (top 5)	
United States of America (%)	16,627 (42.20)
United Kingdom (%)	4084 (10.36)
France (%)	3299 (8.37)
Japan (%)	2086 (5.29)
Italy (%)	1086 (2.76)
(Outcome)	
Life-threatening (%)	1503 (3.81)
Hospitalization - initial or prolonged (%)	8694 (22.06)
Disability (%)	1075 (2.73)
Death (%)	2292 (5.82)
Congenital anomaly (%)	4881 (12.39)
Required intervention to prevent permanent impairment/Damage (%)	331 (0.84)
Other (other serious important medical events) (%)	14,493 (36.78)
Time of occurrence of adverse events - date of medication (days) segment	
0-30d (%)	4921 (12.49)
31-60d (%)	612 (1.55)
61-90d (%)	389 (0.99)
91-120d (%)	298 (0.76)
121-150d (%)	215 (0.55)
151-180d (%)	155 (0.39)
181-360d (%)	1036 (2.63)
>360d (%)	2978 (7.56)
Missing or outlier (less than 0) (%)	28,800 (73.09)
Time of occurrence of adverse events - date of medication (days) (vacancy less than 0)	
N (Missing)	10,604 (28, 800)
Mean (SD)	449.58 (965.26)
Median (Q1, Q3)	45.00 (1.00, 421.50)
Minmax	0.00, 15914.0
Weight (KG)	
N (Missing)	6,901 (32, 503)
Mean (SD)	56.55 (35.03)
Median (Q1, Q3)	62.59 (39.92, 79.38)
Min, max	0.38, 346.99

Annual reporting frequency peaked in 2004 (14.51%) and 2005 (12.56%), followed by variable trends across subsequent years. The majority of reports originated from consumers (55.50%), with additional contributions from physicians (16.70%), other healthcare professionals (8.78%), lawyers (8.15%), and pharmacists (7.59%). Geographically, North America contributed the largest proportion of reports (44.56%), followed by Europe (27.12%) and Asia (6.37%), whereas Oceania, South America, and Africa accounted for 0.39%, 0.21%, and 0.08%, respectively. Country-level analysis indicated that the United States contributed the most reports (42.20%), followed by the United Kingdom (10.36%), France (8.37%), Japan (5.29%), and Italy (2.76%).

Clinical outcome analysis revealed that other serious important medical events constituted the most frequently reported outcomes (36.78%), followed by hospitalizations (22.06%). Reports citing congenital anomalies, death, life-threatening events, disability, and interventions to prevent permanent damage accounted for 12.39%, 5.82%, 3.81%, 2.73%, and 0.84% of cases, respectively. Regarding onset timing, the majority of AEs occurred within 30 days of treatment initiation (12.49%), while 7.56% developed after more than 360 days. Among 6,901 individuals with available weight data, the mean and median body weights were 56.55 kg and 62.59 kg, respectively.

### 3.2 Distribution of the AEs at the organic system class (SOC) level

**FIGURE 2:**
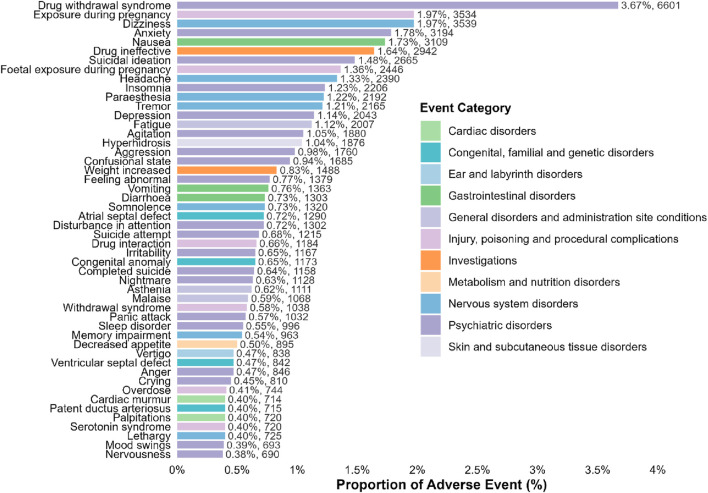
Proportion of signal for PAROXETINE related to AEs at the SOC level.

At the SOC level, a total of 855 PTs met the predefined criteria across all four disproportionality algorithms, indicating positive safety signals associated with paroxetine. The distribution of these signals among SOCs is summarized in Supplementary Table S3 and illustrated in Figure 2. Overall, 26 SOCs were linked to paroxetine-related adverse events, yielding 1,192 distinct positive signals distributed across multiple organ systems.

The most frequently implicated SOC was Various congenital, familial, and genetic disorders, representing 21.90% (261 signals) of all significant signals. Psychiatric disorders constituted the second most common category, accounting for 18.37% (219 signals). Neurological disorders followed, comprising 12.75% (152 signals), suggesting substantial neuropsychiatric involvement in paroxetine’s adverse event profile. Additional SOCs with notable representation included “Investigations” (5.96%, 71 signals), Pregnancy, puerperium, and perinatal conditions (3.69%, 44 signals), Social circumstances (3.61%, 43 signals), and General disorders and administration site conditions (3.36%, 40 signals).

Other relatively frequent SOCs were Cardiac disorders (4.28%, 51 signals), Respiratory, thoracic, and mediastinal disorders (4.11%, 49 signals), and Injury, poisoning, and procedural complications (2.68%, 32 signals). Lower proportions were observed for Gastrointestinal disorders (1.43%), Metabolism and nutrition disorders (1.76%), and Skin and subcutaneous tissue disorders (1.26%).

### 3.3 Distribution of the AEs at the preferred terms level

At the PT level, signal mining conducted using four disproportionality algorithms identified both the most frequent and most strongly associated adverse events related to paroxetine. The overall findings are summarized in Table 2. The most frequently reported PT was Drug withdrawal syndrome, categorized under systemic disorders and administration site reactions, comprising 6,601 cases and exhibiting robust associations across all signal criteria (ROR = 27.27, PRR = 26.31, IC = 4.61, EBGM = 24.37). Other frequently observed neurological PTs included dizziness (3,539 cases) and headache (2,390 cases), each reported at approximately two to three times the expected frequency. Prominent psychiatric manifestations included anxiety (3,194 cases), suicidal ideation (2,665 cases), insomnia (2,206 cases), depression (2,043 cases), and aggression (1,760 cases), all demonstrating statistically significant positive signals.

**TABLE 2:** Top 50 preferred terms of paroxetine by frequency of positive signals for the target drug.

Organ System Classification (SOC)	Preferred Terms (PT)	Case reports (N)	ROR (95% CI)	PRR (Chi-Square)	IC(IC-2SD)	EBGM(EBGM05)
Systemic disease and various reactions at the site of administration	Drug withdrawal syndrome	6,601	27.27 (26.59–27.98)	26.31 (148, 641)	4.61 (4.56)	24.37 (23.76)
Various neurological diseases	Dizzy	3539	2.49 (2.41–2.57)	2.46 (3065.93)	1.29 (1.24)	2.45 (2.37)
All kinds of injuries, poisonings and operational complications	Exposure during pregnancy	3534	13.49 (13.04–13.96)	13.25 (38, 475.9)	3.67 (3.62)	12.76 (12.33)
Psychopathy	Anxiety	3194	3.90 (3.76–4.04)	3.84 (6, 673.06)	1.93 (1.88)	3.81 (3.68)
Diseases of the gastrointestinal system	Disgusting	3109	1.37 (1.33–1.42)	1.37 (310.98)	0.45 (0.40)	1.37 (1.32)
Systemic disease and various reactions at the site of administration	Medications are ineffective	2942	0.77 (0.74–0.80)	0.77 (201.93)	−0.37 (-0.43)	0.77 (0.74)
Psychopathy	Suicidal ideation	2665	10.36 (9.96–10.77)	10.22 (21, 500.3)	3.31 (3.25)	9.93 (9.55)
All kinds of injuries, poisonings and operational complications	Fetal exposure during pregnancy	2446	10.97 (10.54–11.43)	10.84 (21, 145.8)	3.39 (3.33)	10.51 (10.09)
Various neurological diseases	Headache	2390	1.32 (1.27–1.38)	1.32 (183.31)	0.40 (0.34)	1.32 (1.26)
Psychopathy	Insomnia	2206	2.86 (2.74–2.98)	2.84 (2611.67)	1.50 (1.43)	2.82 (2.70)
Various neurological diseases	Abnormal skin sensations	2192	4.84 (4.63–5.05)	4.79 (6, 489.96)	2.24 (2.18)	4.73 (4.54)
Various neurological diseases	Tremor	2165	4.52 (4.34–4.72)	4.48 (5790.24)	2.15 (2.08)	4.43 (4.25)
Psychopathy	Depression	2043	3.06 (2.93–3.20)	3.04 (2782.89)	1.60 (1.53)	3.02 (2.89)
Systemic disease and various reactions at the site of administration	Fatigue	2007	0.90 (0.86–0.94)	0.90 (24.24)	−0.16 (-0.22)	0.90 (0.86)
Psychopathy	Vehement	1880	8.91 (8.50–9.32)	8.82 (12, 702.1)	3.11 (3.03)	8.61 (8.22)
Skin and subcutaneous tissue diseases	Hyperhidrosis	1876	5.06 (4.84–5.30)	5.02 (5962.17)	2.31 (2.24)	4.96 (4.74)
Psychopathy	Aggression	1760	12.39 (11.81–12.99)	12.27 (17, 561.9)	3.57 (3.49)	11.85 (11.30)
Psychopathy	Confusion	1685	3.62 (3.45–3.80)	3.60 (3135.86)	1.84 (1.76)	3.57 (3.40)
All kinds of examinations	Weight gain	1488	2.36 (2.24–2.48)	2.35 (1144.10)	1.22 (1.15)	2.34 (2.22)
Systemic disease and various reactions at the site of administration	Feeling abnormal	1379	1.95 (1.85–2.05)	1.94 (625.23)	0.95 (0.87)	1.93 (1.83)
Diseases of the gastrointestinal system	Vomit	1363	1.01 (0.96–1.07)	1.01 (0.21)	0.02 (-0.06)	1.01 (0.96)
Various neurological diseases	Drowsiness	1320	2.28 (2.16–2.40)	2.27 (931.33)	1.18 (1.09)	2.26 (2.14)
Diseases of the gastrointestinal system	Diarrhea	1303	0.71 (0.67–0.75)	0.71 (158.20)	−0.50 (-0.58)	0.71 (0.67)
Various neurological diseases	Pay attention to obstacles	1302	8.35 (7.90–8.82)	8.29 (8145.74)	3.02 (2.93)	8.11 (7.67)
Various congenital familial genetic disorders	Atrial septal defect	1290	60.58 (57.07–64.31)	60.15 (63, 096.0)	5.66 (5.52)	50.73 (47.79)
Psychopathy	Committing suicidal acts	1215	7.02 (6.63–7.43)	6.98 (6, 094.58)	2.78 (2.69)	6.85 (6.47)
Systemic disease and various reactions at the site of administration	Drug interactions	1184	2.59 (2.45–2.75)	2.58 (1143.21)	1.36 (1.28)	2.57 (2.43)
Various congenital familial genetic disorders	Congenital anomalies	1173	62.86 (59.04–66.93)	62.46 (59, 282.9)	5.71 (5.56)	52.36 (49.17)
Psychopathy	Easily irritated	1167	6.72 (6.34–7.13)	6.69 (5533.51)	2.72 (2.62)	6.57 (6.20)
Psychopathy	Suicide has been completed	1158	4.72 (4.45–5.00)	4.70 (3325.70)	2.22 (2.13)	4.64 (4.38)
Psychopathy	Incubus	1128	11.44 (10.78–12.14)	11.38 (10, 311.8)	3.46 (3.36)	11.02 (10.38)
Systemic disease and various reactions at the site of administration	Weak	1111	1.02 (0.96–1.08)	1.02 (0.25)	0.02 (-0.07)	1.02 (0.96)
Systemic disease and various reactions at the site of administration	Uncomfortable	1068	0.83 (0.78–0.88)	0.83 (38.79)	−0.27 (-0.36)	0.83 (0.78)
Systemic disease and various reactions at the site of administration	Withdrawal syndrome	1038	8.58 (8.06–9.12)	8.53 (6, 725.45)	3.06 (2.96)	8.33 (7.83)
Psychopathy	Panic attacks	1032	9.99 (9.39–10.63)	9.94 (8046.29)	3.27 (3.17)	9.66 (9.08)
Psychopathy	Sleep disturbances	996	5.12 (4.81–5.45)	5.09 (3229.67)	2.33 (2.23)	5.03 (4.72)
Various neurological diseases	Memory impairment	963	2.41 (2.26–2.57)	2.40 (784.74)	1.26 (1.16)	2.39 (2.25)
Metabolic and nutritional diseases	Loss of appetite	895	1.28 (1.19–1.36)	1.27 (52.62)	0.35 (0.25)	1.27 (1.19)
Psychopathy	Wrath	846	8.61 (8.04–9.22)	8.57 (5515.10)	3.07 (2.95)	8.38 (7.82)
Various congenital familial genetic disorders	Ventricular septal defect	842	57.41 (53.34–61.79)	57.15 (39, 368.3)	5.60 (5.42)	48.58 (45.14)
Ear and labyrinthine diseases	Vertigo	838	4.77 (4.46–5.11)	4.75 (2450.43)	2.23 (2.13)	4.70 (4.39)
Systemic disease and various reactions at the site of administration	Cry	810	7.62 (7.11–8.17)	7.59 (4531.24)	2.90 (2.78)	7.44 (6.94)
All kinds of injuries, poisonings and operational complications	Overdose	744	1.13 (1.06–1.22)	1.13 (11.80)	0.18 (0.07)	1.13 (1.05)
Various neurological diseases	Ennui	725	4.35 (4.04–4.68)	4.33 (1835.80)	2.10 (1.99)	4.29 (3.98)
Diseases of the heart organs	Palpitation	720	2.13 (1.98–2.29)	2.13 (428.30)	1.08 (0.97)	2.12 (1.97)
Various neurological diseases	Serotonin syndrome	720	13.82 (12.82–14.89)	13.76 (8171.27)	3.73 (3.59)	13.23 (12.28)
Various congenital familial genetic disorders	Patent ductus arteriosus	715	72.91 (67.21–79.09)	72.62 (41, 106.2)	5.89 (5.66)	59.29 (54.66)
All kinds of examinations	Heart murmur	714	29.98 (27.76–32.37)	29.86 (18, 209.2)	4.78 (4.61)	27.38 (25.36)
Psychopathy	Mood swings	693	7.54 (6.99–8.13)	7.51 (3823.28)	2.88 (2.76)	7.36 (6.83)
Psychopathy	Nervousness	690	4.46 (4.14–4.81)	4.45 (1821.03)	2.14 (2.02)	4.40 (4.08)

Notably, congenital and genetic disorder-related PTs such as atrial septal defect (1,290 cases) and congenital anomaly (1,173 cases) showed markedly elevated signal values (ROR = 60.58 and 62.86, respectively). High-signal events were also evident among PTs associated with exposures during pregnancy, fetal developmental anomalies, and various psychiatric and neurological conditions. Gastrointestinal events (vomiting, diarrhea) and findings categorized under examinations and medical conditions (weight gain, heart murmur) were also represented among the top 50 most frequently reported PTs.

Ranking the top 50 PTs by signal strength according to ROR, PRR, IC, and EBGM metrics (Table 3) revealed several rare but highly disproportionate associations predominantly involving congenital, familial, and genetic disorders. For example, carcinogenic effect on offspring, though documented in only three cases, exhibited the most extreme signal scores (ROR = 952.54, PRR = 952.52, IC = 7.90, EBGM = 238.88), indicating an unusually high reporting frequency relative to baseline expectation. Similarly, PTs such as specific constitutional alcoholism, congenital liver fibrosis, and congenital hyperextension of the spine demonstrated exceptionally elevated signal strengths across all algorithms despite their low occurrence rates. However, the extremely wide confidence interval and small sample size indicate high statistical uncertainty, and such rare signals are particularly susceptible to reporting bias and chance findings. This signal should be interpreted as highly exploratory and requires validation through dedicated epidemiological studies before clinical inference.

**TABLE 3:** Top 50 preferred terms of paroxetine by signal strength of positive signals for the target drug, ranked by EBGM.

Organ System Classification (SOC)	Preferred terms (PT)	Case reports (N)	ROR (95% CI)	PRR (Chi-Square)	IC(IC-2SD)	EBGM(EBGM05)
Various congenital familial genetic disorders	Carcinogenic effect on offspring	3	952.54 (99.08–9157.67)	952.52 (712.89)	7.90 (0.04)	238.88 (24.85)
All kinds of injuries, poisonings and operational complications	Specific constitutional alcoholism	6	635.04 (158.82–2539.23)	635.01 (1266.03)	7.73 (1.34)	212.34 (53.10)
Various congenital familial genetic disorders	Congenital liver fibrosis	3	476.27 (79.58–2850.40)	476.26 (569.12)	7.58 (0.11)	191.10 (31.93)
Various congenital familial genetic disorders	Congenital hyperextension of the spine	4	317.51 (79.41–1269.60)	317.51 (631.02)	7.32 (0.67)	159.25 (39.83)
Various congenital familial genetic disorders	Congenital aortic atresia	22	291.08 (163.22–519.13)	291.05 (3317.81)	7.25 (3.59)	152.33 (85.41)
Various congenital familial genetic disorders	Congenital eye movement impotence	3	238.13 (53.30–1064.03)	238.13 (404.80)	7.09 (0.20)	136.50 (30.55)
Various congenital familial genetic disorders	Congenital aortic stenosis	83	229.26 (172.87–304.05)	229.16 (10, 950.8)	7.06 (5.32)	133.52 (100.67)
Diseases of the heart organs	Subendocardial hemorrhage	3	190.51 (45.53–797.18)	190.50 (353.46)	6.90 (0.23)	119.44 (28.54)
Various congenital familial genetic disorders	Heart-hand syndrome	4	181.44 (53.11–619.81)	181.43 (456.75)	6.86 (0.73)	115.82 (33.90)
Various congenital familial genetic disorders	Marcus gunn syndrome	4	181.44 (53.11–619.81)	181.43 (456.75)	6.86 (0.73)	115.82 (33.90)
Various congenital familial genetic disorders	Congenital mitral stenosis	17	179.94 (99.24–326.24)	179.92 (1930.69)	6.85 (3.17)	115.20 (63.54)
Diseases of the gastrointestinal system	Mucosal prolapse syndrome	5	176.40 (59.12–526.36)	176.39 (560.57)	6.83 (1.12)	113.75 (38.12)
Various congenital familial genetic disorders	Lipid meningocele	7	170.97 (68.21–428.54)	170.97 (768.82)	6.80 (1.71)	111.48 (44.48)
Various congenital familial genetic disorders	Fetal circulation disorder	201	160.13 (135.15–189.72)	159.95 (21, 113.1)	6.74 (5.89)	106.70 (90.06)
Various congenital familial genetic disorders	Congenital aortic regurgitation	43	145.28 (101.27–208.41)	145.24 (4226.39)	6.64 (4.44)	99.97 (69.69)
Various congenital familial genetic disorders	Silla’s syndrome	3	136.08 (35.19–526.24)	136.07 (281.57)	6.58 (0.27)	95.55 (24.71)
Various neurological diseases	Perinatal stroke	10	127.01 (61.00–264.45)	127.00 (892.93)	6.51 (2.31)	91.00 (43.71)
Various congenital familial genetic disorders	The femur is tilted forward	8	127.01 (55.94–288.36)	127.00 (714.35)	6.51 (1.94)	91.00 (40.08)
Various congenital familial genetic disorders	Bicuspid aortic valve	158	124.90 (103.90–150.15)	124.79 (13, 928.2)	6.49 (5.59)	89.86 (74.75)
Various congenital familial genetic disorders	Three chambers of heart	5	122.12 (43.54–342.56)	122.12 (433.79)	6.47 (1.15)	88.47 (31.54)
Various congenital familial genetic disorders	Congenital pulmonary artery stenosis	196	121.92 (103.40–143.75)	121.78 (16, 970.3)	6.46 (5.70)	88.30 (74.89)
Various congenital familial genetic disorders	The tower head is deformed	3	119.07 (31.59–448.82)	119.07 (255.43)	6.44 (0.28)	86.87 (23.04)
Various congenital familial genetic disorders	Maxillofacial bone hypoplasia	4	115.46 (36.76–362.61)	115.46 (332.83)	6.41 (0.77)	84.94 (27.04)
Diseases of the heart organs	Pulmonary stenosis	204	114.97 (97.95–134.95)	114.84 (16, 906.8)	6.40 (5.68)	84.60 (72.08)
Diseases of the heart organs	Subaortic stenosis	9	114.31 (53.36–244.89)	114.30 (743.24)	6.40 (2.14)	84.31 (39.35)
Various congenital familial genetic disorders	Aortic arch dissection	15	113.40 (62.89–204.49)	113.40 (1231.31)	6.39 (2.94)	83.82 (46.48)
Various congenital familial genetic disorders	Congenital aortic anomaly	47	111.39 (79.90–155.30)	111.36 (3805.72)	6.37 (4.46)	82.71 (59.32)
Psychopathy	Thinking insertion	16	103.68 (58.97–182.31)	103.68 (1226.48)	6.29 (3.03)	78.40 (44.59)
Psychopathy	Avoidant personality disorder	11	99.79 (50.68–196.49)	99.79 (818.53)	6.25 (2.45)	76.16 (38.68)
Infections and infectious diseases	Plasmodium vivax infection	5	99.22 (36.35–270.86)	99.22 (370.40)	6.24 (1.16)	75.84 (27.78)
Various congenital familial genetic disorders	Palatal-heart-face syndrome	4	97.70 (31.85–299.63)	97.69 (292.74)	6.23 (0.78)	74.94 (24.44)
Various congenital familial genetic disorders	Congenital tracheomalacia	18	96.88 (57.15–164.22)	96.87 (1308.56)	6.22 (3.19)	74.46 (43.92)
Various neurological diseases	Sympathetic hypersensitivity	3	95.25 (26.21–346.12)	95.25 (215.22)	6.20 (0.30)	73.50 (20.23)
Various congenital familial genetic disorders	Congenital epiglottis abnormalities	3	95.25 (26.21–346.12)	95.25 (215.22)	6.20 (0.30)	73.50 (20.23)
Various congenital familial genetic disorders	Congenital aortic dilatation	5	93.39 (34.45–253.13)	93.38 (353.12)	6.18 (1.16)	72.39 (26.71)
Various congenital familial genetic disorders	Scaphoid deformity	12	90.72 (47.76–172.32)	90.72 (828.12)	6.15 (2.58)	70.78 (37.26)
Various congenital familial genetic disorders	Congenital pulmonary valve stenosis	95	89.29 (71.11–112.11)	89.24 (6, 470.35)	6.13 (5.02)	69.88 (55.65)
Various congenital familial genetic disorders	Craniofacial bone underdevelopment	5	88.20 (32.75–237.56)	88.20 (337.33)	6.11 (1.16)	69.24 (25.71)
Psychopathy	Activation syndrome	73	88.16 (68.03–114.27)	88.13 (4922.10)	6.11 (4.80)	69.20 (53.39)
Various congenital familial genetic disorders	Obstruction of the right ventricular outflow tract	23	86.95 (54.82–137.91)	86.94 (1533.83)	6.10 (3.51)	68.46 (43.16)
Psychopathy	Transvestite	3	86.59 (24.16–310.40)	86.59 (199.42)	6.09 (0.31)	68.25 (19.04)
Various congenital familial genetic disorders	Coarctation of the aorta	199	86.53 (73.97–101.22)	86.43 (13, 209.2)	6.09 (5.45)	68.15 (58.26)
Various congenital familial genetic disorders	Congenital aortic stenosis	31	82.73 (55.72–122.83)	82.71 (1985.28)	6.04 (3.88)	65.82 (44.33)
Various congenital familial genetic disorders	Congenital abdominal hernia	4	79.38 (26.54–237.44)	79.38 (247.65)	5.99 (0.79)	63.70 (21.30)
Various congenital familial genetic disorders	Hypoplastic right heart syndrome	26	77.89 (50.72–119.61)	77.88 (1584.54)	5.97 (3.64)	62.74 (40.85)
Various congenital familial genetic disorders	Ventricular septal defect	15	75.60 (43.05–132.77)	75.60 (891.82)	5.94 (2.89)	61.25 (34.88)
Various congenital familial genetic disorders	Williams syndrome	4	74.71 (25.14–222.03)	74.71 (235.48)	5.92 (0.80)	60.67 (20.41)
Various congenital familial genetic disorders	Congenital flat feet	7	74.09 (32.54–168.68)	74.08 (409.21)	5.91 (1.71)	60.26 (26.47)
Various congenital familial genetic disorders	Congenital pulmonary artery anomalies	19	73.58 (44.67–121.19)	73.57 (1104.21)	5.90 (3.22)	59.92 (36.38)
Various congenital familial genetic disorders	Large vessel misalignment	144	73.33 (61.17–87.90)	73.27 (8340.40)	5.90 (5.15)	59.72 (49.82)

Additionally, several cardiac and vascular malformation-related PTs, including congenital aortic atresia, subendocardial hemorrhage, heart–hand syndrome, and Marcus Gunn syndrome, showed pronounced signal magnitudes, suggesting possible pharmacovigilance relevance to paroxetine exposure. Rare neurological conditions such as perinatal stroke and sympathetic hypersensitivity were also recorded with low case counts but disproportionately strong signal indicators, emphasizing the importance of further clinical evaluation of these associations.

#### 3.3.1 Gender-based subgroup analysis of paroxetine AEs

A gender-stratified disproportionality analysis was performed to assess gender differences in adverse event reporting associated with paroxetine, and PTs meeting all four statistical detection criteria were identified separately for males and females, as shown in Table 4.

**TABLE 4:** Signal strength of paroxetine reports at the preferred term level in the FAERS database grouped by sex.

Sex	Preferred terms (PT)	Case Reports(n)	ROR (95% CI)	PRR (95% CI)	Chi-Sq	IC (IC025)	EBGM (EBGM05)
Female	Atrial septal defect	83	139.2 (111, 174)	136.2 (109, 169)	10,573	7.0 (6.7)	73.1 (61.1)
	Patent ductus arteriosus	31	323.0 (222, 470)	320.4 (221, 465)	8787.0	8.1 (7.6)	52.3 (39.2)
	Ventricular septal defect	32	203.5 (142, 292)	201.8 (141, 289)	5962.6	7.5 (7.0)	48.9 (36.8)
	Foetal exposure during pregnancy	193	47.8 (41.3, 55.3)	45.4 (39.6, 52.2)	8254.3	5.5 (5.3)	40.1 (35.6)
	Cardiac murmur	26	64.5 (43.8, 95.0)	64.0 (43.6, 94.1)	1599.6	6.0 (5.4)	29.2 (21.3)
	Congenital anomaly	18	71.2 (44.8, 113)	70.9 (44.7, 113)	1236.4	6.1 (5.4)	24.7 (17.0)
	Reversible cerebral vasoconstriction syndrome	20	50.7 (32.7, 78.7)	50.5 (32.6, 78.1)	972.3	5.6 (5.0)	23.0 (16.1)
	Heart disease congenital	14	96.7 (57.1, 164)	96.4 (57.0, 163)	1312.9	6.5 (5.8)	22.8 (15.1)
	Serotonin syndrome	33	33.9 (24.1, 47.8)	33.6 (23.9, 47.2)	1045.4	5.1 (4.6)	22.5 (17.0)
	Fallot’s tetralogy	10	232.5 (123, 439)	231.9 (123, 437)	2189.3	7.7 (6.8)	20.0 (12.3)
	Cleft palate	11	108.1 (59.8, 195)	107.8 (59.7, 195)	1161.3	6.7 (5.8)	19.6 (12.3)
	Bicuspid aortic valve	9	607.2 (297, 1243)	605.7 (296, 1238)	4524.4	8.9 (7.9)	19.2 (11.6)
	Antidepressant discontinuation syndrome	9	463.5 (231, 932)	462.4 (230, 929)	3631.9	8.6 (7.6)	19.1 (11.5)
	Pulmonary valve stenosis	9	426.8 (213, 854)	425.8 (213, 851)	3388.5	8.5 (7.5)	19.0 (11.5)
	Talipes	10	132.8 (71.2, 248)	132.4 (71.1, 246)	1293.6	7.0 (6.1)	18.8 (11.6)
	Withdrawal syndrome	61	21.0 (16.3, 27.0)	20.6 (16.1, 26.5)	1139.8	4.4 (4.0)	17.7 (14.4)
	Pulmonary artery stenosis congenital	8	397.5 (192, 825)	396.7 (192, 822)	2854.5	8.4 (7.4)	17.1 (10.1)
	Congenital central nervous system anomaly	8	228.2 (113, 462)	227.7 (113, 461)	1743.0	7.7 (6.7)	16.6 (9.8)
	Inappropriate antidiuretic hormone secretion	18	24.7 (15.6, 39.1)	24.6 (15.6, 38.8)	413.8	4.6 (3.9)	15.1 (10.4)
	Hyponatraemia	62	17.3 (13.5, 22.2)	17.0 (13.3, 21.8)	937.7	4.1 (3.7)	15.0 (12.2)
Male	Atrial septal defect	90	174.6 (141, 217)	167.9 (136, 207)	14,063	7.3 (7.0)	84.6 (71.3)
	Foetal exposure during pregnancy	312	81.8 (72.5, 92.3)	71.0 (63.9, 78.9)	21,006	6.1 (6.0)	62.3 (56.8)
	Talipes	35	339.5 (239, 483)	334.3 (236, 474)	10,393	8.2 (7.7)	58.0 (44.2)
	Cardiac murmur	42	178.2 (130, 244)	175.0 (129, 238)	6,865.9	7.4 (6.9)	56.6 (44.1)
	Patent ductus arteriosus	30	430.4 (292, 633)	424.8 (290, 622)	11,004	8.5 (8.0)	53.1 (39.6)
	Ventricular septal defect	31	217.7 (151, 314)	214.8 (150, 308)	6,168.3	7.6 (7.1)	48.5 (36.4)
	Cleft palate	20	333.1 (210, 529)	330.1 (209, 523)	5934.7	8.2 (7.5)	36.8 (25.9)
	Cleft lip and palate	16	259.2 (156, 431)	257.3 (155, 427)	3816.1	7.9 (7.1)	29.8 (20.1)
	Congenital anomaly	22	79.1 (51.9, 121)	78.4 (51.6, 119)	1666.6	6.2 (5.6)	28.9 (20.6)
	Heart disease congenital	16	130.2 (79.2, 214)	129.3 (78.9, 212)	1996.1	6.9 (6.2)	26.8 (18.1)
	Attention deficit/hyperactivity disorder	19	76.8 (48.9, 121)	76.2 (48.7, 119)	1404.3	6.2 (5.6)	26.2 (18.2)
	Poisoning deliberate	22	56.3 (37.0, 85.7)	55.8 (36.8, 84.5)	1183.6	5.8 (5.2)	25.2 (18.0)
	Fallot’s tetralogy	12	385.9 (212, 701)	383.9 (212, 696)	4131.8	8.4 (7.5)	24.2 (15.5)
	Autism spectrum disorder	24	42.1 (28.2, 62.9)	41.7 (28.0, 62.0)	956.6	5.4 (4.8)	22.8 (16.5)
​	Serotonin syndrome	27	38.0 (26.0, 55.5)	37.5 (25.8, 54.6)	964.0	5.2 (4.7)	22.5 (16.6)
	Bicuspid aortic valve	11	383.5 (206, 714)	381.6 (205, 709)	3781.7	8.4 (7.5)	22.4 (14.1)
	Autism	11	114.0 (63.1, 206)	113.5 (62.9, 205)	1226.0	6.8 (5.9)	19.8 (12.5)
	Loss of libido	23	30.4 (20.2, 45.7)	30.1 (20.1, 45.1)	652.5	4.9 (4.3)	18.6 (13.4)
	Coarctation of the aorta	9	272.6 (139, 533)	271.5 (139, 530)	2307.0	7.9 (7.0)	18.6 (11.2)
	Genital anaesthesia	9	244.4 (125, 476)	243.4 (125, 473)	2087.5	7.8 (6.8)	18.4 (11.1)

​Among female reports, the strongest signals were for congenital cardiac and pregnancy-related outcomes, including atrial septal defect, ventricular septal defect, patent ductus arteriosus, cardiac murmur, congenital anomaly, heart disease congenital, and foetal exposure during pregnancy. The highest EBGM values were observed for atrial septal defect (EBGM = 73.1) and patent ductus arteriosus (EBGM = 52.3), indicating strong signal strength within the female subgroup (Table 4).

​Among male reports, a broadly similar pattern was seen, with pronounced disproportionality for atrial septal defect, ventricular septal defect, patent ductus arteriosus, cardiac murmur, talipes, and foetal exposure during pregnancy; the highest EBGM values were observed for atrial septal defect (EBGM = 84.6) and foetal exposure during pregnancy (EBGM = 62.3) (Table 4).

#### 3.3.2 Age-based subgroup analysis

An age-stratified disproportionality analysis was performed to identify age-specific adverse event signals associated with paroxetine, with PTs meeting all four statistical detection criteria summarized by age group in Table 5.

**TABLE 5:** Signal strength of paroxetine at the preferred term level grouped by age.

Age	Preferred terms (PT)	Case reports(n)	ROR (95% CI)	PRR (95% CI)	Chi-Sq	IC (IC025)	EBGM (EBGM05)
<18	Foetal exposure during pregnancy	126	221.9 (176, 280)	129.7 (113, 149)	15,048	6.9 (6.7)	82.1 (71.0)
	Atrial septal defect	48	509.3 (363, 714)	427.9 (319, 574)	16,472	8.4 (8.0)	76.4 (60.5)
	Patent ductus arteriosus	22	1602 (898, 2858)	1483 (851, 2585)	17,704	9.6 (8.8)	43.5 (31.0)
	Ventricular septal defect	17	542.8 (314, 939)	511.5 (303, 863)	6,837.2	8.6 (7.9)	33.1 (22.6)
	Talipes	16	484.7 (278, 846)	458.4 (269, 781)	5917.6	8.5 (7.7)	31.2 (21.1)
	Cardiac murmur	14	282.8 (161, 498)	269.3 (156, 463)	3341.2	7.9 (7.1)	26.7 (17.6)
18-44	Reversible cerebral vasoconstriction syndrome	13	46.1 (26.8, 79.5)	45.7 (26.6, 78.3)	571.9	5.5 (4.7)	17.4 (11.3)
	Poisoning deliberate	20	23.7 (15.3, 36.8)	23.4 (15.1, 36.0)	432.0	4.5 (3.9)	15.1 (10.6)
	Alcohol abuse	11	34.3 (19.1, 61.7)	34.0 (19.0, 60.9)	360.0	5.1 (4.2)	14.2 (8.9)
	Anorgasmia	13	28.1 (16.4, 48.3)	27.9 (16.3, 47.6)	343.0	4.8 (4.0)	14.1 (9.2)
	Serotonin syndrome	19	20.9 (13.3, 32.8)	20.6 (13.2, 32.1)	359.0	4.3 (3.7)	13.7 (9.5)
	Withdrawal syndrome	26	15.1 (10.3, 22.2)	14.8 (10.1, 21.7)	338.6	3.9 (3.3)	11.7 (8.6)
	Sexual dysfunction	16	15.7 (9.7, 25.7)	15.6 (9.6, 25.2)	222.7	3.9 (3.3)	10.8 (7.3)
	Libido decreased	17	15.3 (9.5, 24.6)	15.1 (9.5, 24.1)	228.2	3.9 (3.2)	10.8 (7.4)
	Hallucination, auditory	13	16.9 (9.9, 29.0)	16.8 (9.8, 28.6)	197.8	4.1 (3.3)	10.7 (6.9)
	Erectile dysfunction	25	12.5 (8.4, 18.5)	12.3 (8.4, 18.1)	262.7	3.6 (3.1)	10.0 (7.3)
	Loss of libido	19	12.7 (8.1, 19.8)	12.5 (8.0, 19.4)	204.5	3.6 (3.0)	9.6 (6.7)
45-64	Serotonin syndrome	15	35.4 (21.4, 58.6)	35.0 (21.3, 57.7)	505.0	5.1 (4.4)	16.9 (11.3)
	Reversible cerebral vasoconstriction syndrome	10	54.2 (29.3, 100)	53.8 (29.3, 98.9)	532.2	5.7 (4.8)	15.7 (9.7)
	Sopor	10	27.1 (14.7, 50.0)	26.9 (14.7, 49.4)	259.5	4.7 (3.9)	12.3 (7.6)
	Withdrawal syndrome	16	18.5 (11.4, 30.2)	18.3 (11.3, 29.6)	268.2	4.2 (3.5)	12.1 (8.2)
	Erectile dysfunction	16	16.6 (10.2, 26.9)	16.4 (10.1, 26.5)	236.6	4.0 (3.3)	11.2 (7.6)
	Suicide attempt	15	15.1 (9.1, 24.9)	14.9 (9.1, 24.5)	200.3	3.9 (3.2)	10.4 (6.9)
	Hyponatraemia	16	13.8 (8.5, 22.4)	13.6 (8.4, 22.0)	191.8	3.8 (3.1)	9.9 (6.7)
	Electrocardiogram qt prolonged	15	13.8 (8.3, 22.8)	13.6 (8.3, 22.4)	180.5	3.8 (3.0)	9.7 (6.5)
>64	Serotonin syndrome	22	94.0 (61.5, 144)	92.1 (60.8, 140)	1969.0	6.5 (5.9)	30.6 (21.8)
	Haematoma muscle	11	116.8 (64.6, 211)	115.6 (64.3, 208)	1258.6	6.8 (6.0)	19.9 (12.5)
	Hyponatraemia	59	22.8 (17.6, 29.6)	21.7 (16.9, 27.8)	1168.2	4.4 (4.1)	18.4 (14.9)
	Inappropriate antidiuretic hormone secretion	15	26.1 (15.8, 43.2)	25.8 (15.7, 42.3)	366.2	4.7 (4.0)	14.5 (9.7)
	Haemorrhagic stroke	10	24.5 (13.3, 45.1)	24.3 (13.3, 44.4)	232.5	4.6 (3.7)	11.8 (7.2)
	Altered state of consciousness	18	14.1 (8.9, 22.3)	13.9 (8.8, 21.8)	220.0	3.8 (3.1)	10.3 (7.1)
	Hepatic cytolysis	10	14.6 (8.0, 26.9)	14.5 (7.9, 26.5)	131.4	3.9 (3.0)	9.0 (5.5)

Among patients younger than 18 years, the strongest signals were consistently for foetal exposure during pregnancy and congenital malformations, particularly cardiac defects such as atrial septal defect (EBGM = 76.4), patent ductus arteriosus (EBGM = 43.5), ventricular septal defect (EBGM = 33.1), along with talipes (EBGM = 31.2) and cardiac murmur (EBGM = 26.7).

In adults aged 18–44 years, signals shifted toward reversible cerebral vasoconstriction syndrome (EBGM = 17.4), deliberate poisoning (EBGM = 15.1), serotonin syndrome (EBGM = 13.7), withdrawal syndrome (EBGM = 11.7), and multiple sexual dysfunctions including anorgasmia (EBGM = 14.1), Qsexual dysfunction (EBGM = 10.8), libido decreased (EBGM = 10.8), erectile dysfunction (EBGM = 10.0), and loss of libido (EBGM = 9.6).

​For the 45–64-year age group, prominent PTs included serotonin syndrome (EBGM = 16.9), reversible cerebral vasoconstriction syndrome (EBGM = 15.7), withdrawal syndrome (EBGM = 12.1), erectile dysfunction (EBGM = 11.2), suicide attempt (EBGM = 10.4), hyponatraemia (EBGM = 9.9), and electrocardiogram QT prolongation (EBGM = 9.7), indicating continued neuropsychiatric and cardiovascular concerns.

​In patients over 64 years, serotonin syndrome showed the highest signal strength (EBGM = 30.6), followed by muscle hematoma (EBGM = 19.9), hyponatraemia (EBGM = 18.4), inappropriate antidiuretic hormone secretion (EBGM = 14.5), hemorrhagic stroke (EBGM = 11.8), and altered state of consciousness (EBGM = 10.3), reflecting pronounced risks in older adults.

#### 3.3.3 Paroxetine pregnancy and fetal subgroup analysis

A pregnancy-specific disproportionality analysis was conducted to identify signals associated with paroxetine exposure during pregnancy, with PTs meeting all four statistical detection criteria summarized in Table 6.

**TABLE 6:** Pregnancy and fetal-related safety events of paroxetine at PT level.

Preferred term (PT)	Case reports	ROR (95% CI)	PRR (95% CI)	Chi-square	IC (IC025)	EBGM (EB05)
Persistent foetal circulation	4	49.82 (18.55, 133.77)	49.05 (18.52, 129.90)	188.06453	5.45 (4.04)	8.30 (4.12)
Foetal exposure during pregnancy	194	22.29 (17.37, 28.60)	7.81 (7.20, 8.48)	1242.2464	2.94 (2.82)	7.57 (6.73)
Foetal movement disorder	2	114.81 (27.89, 472.60)	113.81 (27.92, 464.03)	216.04324	6.46 (4.43)	5.68 (2.36)
Bradycardia foetal	3	11.86 (4.07, 34.57)	11.73 (4.07, 33.75)	33.366358	3.51 (1.99)	4.96 (2.29)
Tachycardia foetal	1	14.06 (2.75, 71.96)	13.99 (2.75, 71.02)	17.463275	3.76 (1.41)	3.28 (1.16)
Twin pregnancy	2	6.11 (1.74, 21.43)	6.07 (1.75, 21.04)	10.427544	2.58 (0.79)	3.27 (1.36)
Abortion of ectopic pregnancy	1	7.15 (1.42, 36.12)	7.12 (1.42, 35.66)	7.7569352	2.81 (0.49)	2.80 (0.99)
Foetal heart rate deceleration abnormality	1	6.04 (1.20, 30.43)	6.02 (1.20, 30.05)	6.1814168	2.57 (0.25)	2.66 (0.94)
Foetal distress syndrome	1	2.42 (0.48, 12.11)	2.41 (0.49, 11.97)	1.2367857	1.27 (−1.04)	1.78 (0.63)
Foetal growth restriction	1	0.68 (0.14, 3.39)	0.68 (0.14, 3.37)	0.2242935	−0.55 (−2.86)	0.74 (0.26)
Maternal exposure during breast feeding	2	0.60 (0.17, 2.10)	0.61 (0.18, 2.09)	0.6455941	−0.72 (−2.50)	0.65 (0.27)
Exposure during pregnancy	54	0.49 (0.37, 0.66)	0.59 (0.46, 0.75)	23.031953	−0.76 (−1.11)	0.59 (0.48)
Foetal death	1	0.29 (0.06, 1.43)	0.29 (0.06, 1.44)	2.6337484	−1.78 (−4.08)	0.36 (0.13)
Maternal exposure during pregnancy	29	0.25 (0.17, 0.36)	0.32 (0.23, 0.46)	61.124698	−1.63 (−2.12)	0.33 (0.24)
Pregnancy	1	0.08 (0.02, 0.41)	0.09 (0.02, 0.43)	15.255426	−3.52 (−5.82)	0.11 (0.04)

​The strongest signal was observed for persistent foetal circulation (EBGM = 8.30), followed by foetal exposure during pregnancy, which had the highest case volume (EBGM = 7.57). ​Other prominent pregnancy- and foetal disorder-related signals included foetal movement disorder (EBGM = 5.68), bradycardia foetal (EBGM = 4.96), tachycardia foetal (EBGM = 3.28), twin pregnancy (EBGM = 3.27), abortion of ectopic pregnancy (EBGM = 2.80), and foetal heart rate deceleration abnormality (EBGM = 2.66).

### 3.4 ​Time-to-onset analysis

**FIGURE 3:**
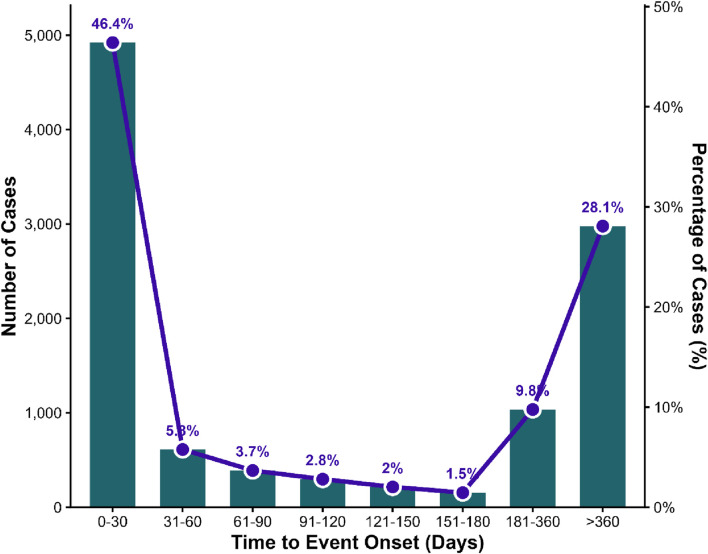
Onset time distribution of paroxetine-related adverse events (AEs).

Among the 39,404 AE reports associated with paroxetine, 10,604 cases contained complete information suitable for time-to-onset analysis. As illustrated in Figure 3, the distribution of onset times exhibited a markedly right-skewed pattern, indicating that most AEs occurred shortly after treatment initiation. The 0–30-day interval represented the largest proportion of cases (46.41%, n = 4,921), while events occurring beyond 360 days accounted for 28.08% (n = 2,978). Collectively, these two categories comprised nearly three-quarters of all AEs with reported onset information. Intermediate onset intervals contributed smaller proportions: 31–60 days (5.77%), 61–90 days (3.67%), 91–120 days (2.81%), 121–150 days (2.03%), 151–180 days (1.46%), and 181–360 days (9.77%).

### 3.5 Comparative analysis of paroxetine vs. sertraline

To differentiate class effects from paroxetine-specific risks, a comparative analysis was performed using sertraline as a reference. While both SSRIs show high reporting volumes for psychiatric symptoms, their signal intensities diverge significantly in specific domains. As shown in Supplementary Table S4, notable differences were observed between paroxetine and sertraline in both the distribution of PTs and the corresponding signal strengths. Furthermore, paroxetine exhibits a disproportionately higher intensity for rare congenital malformations, such as congenital aortic atresia, whereas sertraline signals in this SOC are generally lower in magnitude, suggesting a drug-specific rather than class-wide vulnerability.

## 4 Discussion

In this large-scale post-marketing pharmacovigilance study of paroxetine using the FAERS database, both established and emerging safety signals were identified across diverse adverse event categories. Application of multiple disproportionality algorithms enabled robust detection of potential drug event associations. As expected, drug withdrawal syndrome emerged as a major signal, consistent with the well-documented discontinuation effects of paroxetine. Previous clinical evidence indicates that abrupt cessation or rapid dose reduction can elicit physical and psychological symptoms such as dizziness, sensory disturbances, irritability, and sleep (Fava and Cosci, 2019; Lader, 2007; Henssler et al., 2024). The high incidence of antidepressant discontinuation symptoms reported in several population-based clinical studies further supports these findings, highlighting paroxetine’s relatively short half-life and strong serotonin reuptake inhibition as key contributors to its elevated withdrawal risk among SSRIs (Kalfas et al., 2025).

The analysis also revealed notable signals involving congenital, familial, and genetic disorders, raising concerns regarding paroxetine use during pregnancy. It is critical to note that rare signals with very small case count (carcinogenic effect on offspring, congenital liver fibrosis) demonstrate extreme statistical disproportionality but have wide confidence intervals and high susceptibility to reporting bias. These should be considered exploratory findings requiring urgent validation rather than established risks. While spontaneous reporting data cannot infer causality, the disproportionate reporting of these events is consistent with epidemiologic and meta-analytic evidence indicating that first-trimester exposure to paroxetine may slightly elevate the risk of congenital anomalies, particularly cardiac malformations (Bérard et al., 2016; Bracken, 2016; Huybrechts et al., 2018). Several large-scale studies have demonstrated a statistically significant association between early paroxetine exposure and septal defects, although the absolute risk remains low relative to background incidence (Reefhuis et al., 2015; Ross et al., 2013). These findings highlight the need for individualized risk-benefit evaluation when prescribing paroxetine to women of reproductive age and emphasize vigilant monitoring and counseling on potential developmental risks. Clinicians should consider alternative therapies when feasible and coordinate with obstetric and perinatal care specialists to ensure maternal and fetal safety. Several rare congenital signals identified in our analysis warrant cautious interpretation. While signals such as carcinogenic effect on offspring and congenital liver fibrosis demonstrate extreme statistical disproportionality, their clinical significance remains uncertain due to very small sample sizes and potential reporting bias inherent in spontaneous reporting systems. These rare events may be more likely to be reported precisely because of their severity or unexpectedness, inflating signal strength metrics. Cross-reference with existing epidemiological literature reveals limited biological plausibility for some of these associations, particularly carcinogenic effects, which lack mechanistic support in preclinical or clinical studies. These signals should serve as hypothesis-generating alerts for further investigation through well-designed cohort studies with adequate statistical power, rather than being interpreted as confirmed risks.

Another notable pattern identified in this analysis was the temporal distribution of adverse events, characterized by clusters occurring either shortly after treatment initiation or following prolonged exposure. This suggests that paroxetine may contribute not only to acute adaptation or withdrawal-related reactions but also to delayed or cumulative toxicities. Such effects may encompass psychiatric, neurological, metabolic, or cardiovascular complications, which are often underrepresented in controlled clinical trials due to their limited duration and selective enrollment criteria (Fabian et al., 2003; Edinoff et al., 2021; Dannon et al., 2004). These findings underscore the importance of continuous pharmacovigilance and long-term monitoring, particularly among patients maintained on chronic therapy, to facilitate early detection and management of late-onset adverse outcomes.

The safety profile of paroxetine in high-risk populations requires careful evaluation. Older adults and patients with multiple comorbidities appear especially vulnerable to adverse outcomes such as hyponatremia, anticholinergic effects, and drug–drug interactions. Hyponatremia has been frequently documented among elderly individuals receiving SSRIs, including paroxetine, with low body weight and advanced age identified as key risk factors (Wilkinson et al., 1999). Furthermore, paroxetine’s pharmacologic characteristics, particularly its strong inhibition of CYP2D6 and influence on cardiac ion channels, necessitate heightened vigilance in cases involving polypharmacy or underlying cardiovascular disease (Nevels et al., 2016; Plijter et al., 2023). The high-intensity signals likely arise from paroxetine’s potent serotonin transporter (SERT) inhibition and strong CYP2D6 inhibition. Unlike other SSRIs, paroxetine’s CYP2D6 inhibition causes autoinhibition of its own metabolism and significant drug-drug interactions, explaining the signal diversity observed here. Elevated cardiac malformation signals may reflect paroxetine’s disruption of serotonin-mediated pathways essential for embryonic heart development and fetal cardiogenesis. Clinicians should incorporate these factors into individualized treatment planning and apply targeted monitoring to minimize potential safety concerns.

The psychiatric and neurological adverse events observed, such as anxiety, insomnia, suicidal ideation, and worsening or new-onset depressive symptoms, likely reflect both disease-related and drug-induced effects (Montgomery, 1993). Meta-analyses of clinical trials found no overall increase in suicidality compared with placebo, though a higher incidence was observed among younger adults and specific diagnostic subgroups (Carpenter et al., 2011). These findings emphasize the need for thorough baseline evaluation, continuous monitoring, and prompt management of emerging psychiatric symptoms. Most reported events align with the established safety profile of paroxetine (De Crescenzo et al., 2025). Yet, spontaneous reporting also identified potential underrecognized outcomes, highlighting the complementary role of large-scale pharmacovigilance in refining safety assessment beyond controlled trials.

From a clinical standpoint, the findings offer several practical implications for optimizing the safe and effective use of paroxetine. Implementation of structured tapering protocols, close monitoring during treatment initiation, cautious use during pregnancy, and continuous evaluation of long-term safety are essential components of risk management. Moreover, recognizing high-risk groups, such as older adults and individuals with comorbidities, can support personalized prescribing decisions and enhance overall patient safety (Horowitz and Taylor, 2019; Romdhani et al., 2023).

Our findings corroborate and extend prior pharmacovigilance research on paroxetine. The withdrawal syndrome signal aligns with meta-analytic evidence of 30%–56% discontinuation symptoms and exceeds earlier FAERS analyses, reflecting 21 years of accumulated data and heightened awareness (Chiappini et al., 2022; Henssler et al., 2024). Cardiac malformation signals substantially exceed population-based meta-analyses, consistent with FAERS enrichment for severe outcomes (Bérard et al., 2016). Critically, comparative analysis with sertraline revealed paroxetine-specific elevations distinguishing drug-from indication-driven effects.

## 5 Conclusion

This FAERS-based signal detection analysis identified a consistent adverse event profile for paroxetine, primarily characterized by neuropsychiatric reactions, including withdrawal-related and suicidality-associated reports, as well as a distinct cluster of congenital and perinatal signals linked to pregnancy exposure. The temporal distribution revealed that most adverse events occurred during the initial month of therapy, emphasizing this period as a critical phase for monitoring, while a secondary cluster emerged during long-term treatment. However, these findings must be interpreted within the inherent limitations of spontaneous reporting, including bias and the inability to establish definitive causality. Suggesting potential risks associated with chronic exposure or delayed manifestation. Overall, the findings reaffirm established safety concerns while highlighting additional signals that merit further investigation. In clinical practice, priority should be given to tailored patient selection, structured dose tapering to prevent acute withdrawal, pregnancy risk counseling, and vigilant monitoring of cardiac health in infants exposed to utero. These strategies are crucial to enhancing the safety and therapeutic outcomes of paroxetine use in real-world settings.

## 6 Limitation

While analyses were restricted to paroxetine as the primary suspect drug, residual confounding from unmeasured factors (disease severity, dose-response, treatment duration, polypharmacy) cannot be excluded. FAERS lack structured data for multivariable adjustment. However, comparison with sertraline, sharing identical indications, revealed paroxetine-specific signals, indicating drug-specific rather than indication-driven effects. Causal inference requires pharmacoepidemiologic studies with complete clinical covariates.

## Data Availability

The original contributions presented in the study are included in the article/Supplementary Material, further inquiries can be directed to the corresponding authors.
