# Supplementary material for: Post-marketing safety evaluation of paroxetine: a real-world pharmacovigilance analysis based on the FDA adverse event reporting system
Source: Front Pharmacol. 2026 Apr 14;17:1744180. doi: 10.3389/fphar.2026.1744180 (PMC13121166; doi:10.3389/fphar.2026.1744180)
Supplement: Supplementary file 1 [file Table1.docx]

**Sup table 1.** The 2x2 contingency table for disproportionality analysis.

|  | **Target Adverse Event Cases** | **Other Adverse Event Cases** | **Total** |
| --- | --- | --- | --- |
| Target Drug | A | b | a+b |
| Other Drugs | C | d | c+d |
| Total | A+C | b+d | N=a+b+c+d |

a: Number of reports for paroxetine involving the target adverse event.

b: Number of reports for paroxetine involving all other adverse events.

c: Number of reports for all other drugs involving the target adverse event.

d: Number of reports for all other drugs involving all other adverse events.

n: The total number of reports in the database (n = a + b + c + d).

**Sup table 2.** Signal detection algorithms, formulas, and established thresholds used in the analysis.

| **Algorithms** | **Equation** | **Threshold** |
| --- | --- | --- |
| ROR | ROR = $\frac{ad}{bc}$  $SE(lnROR) = \sqrt{\left( \frac{1}{a}+\frac{1}{b}+\frac{1}{c}+\frac{1}{d} \right)}$ 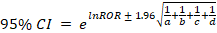 | N ≥ 3, lower limit of 95% CI > 1 |
| PRR | $PRR =\frac{\frac{a}{a+b}}{\frac{c}{c+d}}$   $SE(lnPRR) = \sqrt{\frac{1}{a}-\frac{1}{a+b}+\frac{1}{c}-\frac{1}{c+d}}$ 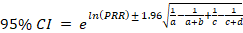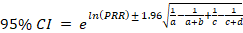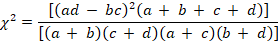 | N ≥ 3, lower limit of 95% CI > 1, χ² ≥ 4 |
| BCPNN | $95\% CI = E(IC) \pm2\sqrt{V\left( IC \right)}$ 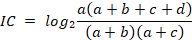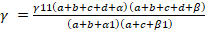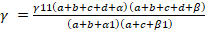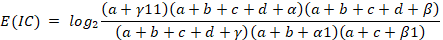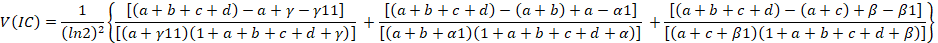 | IC025 > 0 |
| EBGM | $EBGM =\frac{a+b+c+d}{\left( a+c \right)\left( a+b \right)}$ 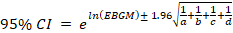 | EBGM05 > 2 |

a: Number of reports for paroxetine involving the target adverse event.

b: Number of reports for paroxetine involving all other adverse events.

c: Number of reports for all other drugs involving the target adverse event.

d: Number of reports for all other drugs involving all other adverse events.

n: The total number of reports in the database (n = a + b + c + d).

**Sup table 3.** Distribution of positive signals of adverse events associated with the paroxetine across system organ classes

| **Organ system classification** | **Number of positive signals** | **Proportion (%)** |
| --- | --- | --- |
| Psychopathy | 219 | 18.37 |
| Various neurological diseases | 152 | 12.75 |
| Systemic disease and various reactions at the site of administration | 40 | 3.36 |
| All kinds of injuries, poisonings and operational complications | 32 | 2.68 |
| Various congenital familial genetic disorders | 261 | 21.90 |
| Diseases of the gastrointestinal system | 17 | 1.43 |
| All kinds of examinations | 71 | 5.96 |
| Diseases of the heart organs | 51 | 4.28 |
| Skin and subcutaneous tissue diseases | 15 | 1.26 |
| Respiratory, thoracic and mediastinal diseases | 49 | 4.11 |
| Various musculoskeletal and connective tissue diseases | 26 | 2.18 |
| Metabolic and nutritional diseases | 21 | 1.76 |
| Diseases of the eye organs | 28 | 2.35 |
| Vascular and lymphatic diseases | 20 | 1.68 |
| Ear and labyrinthine diseases | 8 | 0.67 |
| Infections and infectious diseases | 12 | 1.01 |
| social environment | 43 | 3.61 |
| Reproductive system and breast diseases | 25 | 2.10 |
| Pregnancy, puerperium, and perinatal conditions | 44 | 3.69 |
| Kidney and urinary system diseases | 18 | 1.51 |
| Diseases of the hepatobiliary system | 9 | 0.76 |
| Blood and lymphatic system diseases | 12 | 1.01 |
| Product Questions | 2 | 0.17 |
| Various surgeries and medical procedures | 6 | 0.50 |
| Diseases of the endocrine system | 7 | 0.59 |
| Immune system disorders | 1 | 0.08 |
| Benign, malignant, and tumors of unknown nature (including cystic and polyp-like) | 3 | 0.25 |
| Total | 1192 | 100.00 |

**Sup table 4.** Signal strength of Sertraline-associated adverse events at the Preferred Term level ranked by number of reports

| **System Organ Class (SOC)** | **Preferred Term (PT)** | **Case reports** | **ROR (95% CI)** | **PRR (95% CI)** | **Chi Square** | **IC (IC025)** | **EBGM (EBGM05)** |
| --- | --- | --- | --- | --- | --- | --- | --- |
| Psychiatric disorders | Anxiety | 1499 | 4.48  (4.26,4.72) | 4.41  (4.20,4.64) | 3951.84 | 2.14  (2.06) | 4.39  (4.17) |
| Psychiatric disorders | Insomnia | 1142 | 3.64  (3.43,3.86) | 3.60  (3.40,3.81) | 2141.26 | 1.84  (1.75) | 3.59  (3.38) |
| Psychiatric disorders | Suicidal ideation | 1012 | 9.47  (8.89,10.08) | 9.35  (8.79,9.95) | 7471.82 | 3.21  (3.11) | 9.26  (8.70) |
| General disorders and administration site conditions | Feeling abnormal | 871 | 3.04  (2.84,3.25) | 3.02  (2.82,3.22) | 1174.17 | 1.59  (1.49) | 3.01  (2.81) |
| Psychiatric disorders | Depression | 868 | 3.20  (2.99,3.42) | 3.18  (2.97,3.39) | 1293.57 | 1.66  (1.56) | 3.17  (2.96) |
| Nervous system disorders | Tremor | 844 | 4.30  (4.01,4.60) | 4.26  (3.98,4.56) | 2099.90 | 2.08  (1.98) | 4.24  (3.96) |
| Immune system disorders | Drug hypersensitivity | 592 | 2.58  (2.38,2.80) | 2.57  (2.37,2.79) | 568.55 | 1.36  (1.24) | 2.57  (2.37) |
| Product issues | Product substitution issue | 534 | 7.91  (7.26,8.62) | 7.86  (7.22,8.56) | 3169.50 | 2.96  (2.82) | 7.79  (7.16) |
| Injury, poisoning and procedural complications | Maternal exposure during pregnancy | 534 | 5.37  (4.93,5.85) | 5.34  (4.91,5.82) | 1874.83 | 2.41  (2.27) | 5.31  (4.88) |
| Psychiatric disorders | Abnormal dreams | 533 | 16.21  (14.87,17.67) | 16.10  (14.78,17.54) | 7403.24 | 3.98  (3.82) | 15.80  (14.50) |
| Psychiatric disorders | Nightmare | 503 | 12.34  (11.30,13.48) | 12.26  (11.23,13.39) | 5128.06 | 3.60  (3.43) | 12.09  (11.07) |
| Skin and subcutaneous tissue disorders | Hyperhidrosis | 500 | 3.26  (2.99,3.56) | 3.25  (2.98,3.54) | 776.33 | 1.70  (1.56) | 3.24  (2.97) |
| Psychiatric disorders | Suicide attempt | 500 | 6.99  (6.40,7.64) | 6.95  (6.37,7.59) | 2527.69 | 2.79  (2.64) | 6.90  (6.32) |
| Cardiac disorders | Palpitations | 476 | 3.45  (3.15,3.77) | 3.43  (3.14,3.75) | 818.65 | 1.77  (1.63) | 3.42  (3.13) |
| Psychiatric disorders | Agitation | 475 | 5.41  (4.94,5.92) | 5.38  (4.92,5.89) | 1685.16 | 2.42  (2.27) | 5.35  (4.89) |
| Psychiatric disorders | Irritability | 465 | 6.51  (5.94,7.14) | 6.48  (5.91,7.09) | 2138.37 | 2.69  (2.53) | 6.43  (5.87) |
| General disorders and administration site conditions | Withdrawal syndrome | 465 | 9.31  (8.49,10.20) | 9.26  (8.45,10.14) | 3388.57 | 3.20  (3.04) | 9.16  (8.36) |
| Nervous system disorders | Disturbance in attention | 448 | 6.97  (6.35,7.66) | 6.94  (6.32,7.61) | 2258.86 | 2.78  (2.63) | 6.89  (6.27) |
| Injury, poisoning and procedural complications | Foetal exposure during pregnancy | 437 | 4.59  (4.18,5.04) | 4.57  (4.16,5.02) | 1212.90 | 2.19  (2.04) | 4.55  (4.14) |
| Nervous system disorders | Paraesthesia | 423 | 2.25  (2.05,2.48) | 2.25  (2.04,2.47) | 292.45 | 1.17  (1.02) | 2.24  (2.04) |
| Psychiatric disorders | Depressed mood | 415 | 6.75  (6.13,7.44) | 6.72  (6.10,7.40) | 2004.81 | 2.74  (2.58) | 6.67  (6.05) |
| Psychiatric disorders | Apathy | 393 | 23.49  (21.24,25.97) | 23.37  (21.14,25.83) | 8177.87 | 4.51  (4.28) | 22.73  (20.56) |
| General disorders and administration site conditions | Crying | 384 | 8.85  (8.01,9.79) | 8.81  (7.97,9.74) | 2632.80 | 3.13  (2.95) | 8.73  (7.89) |
| Psychiatric disorders | Panic attack | 367 | 8.52  (7.69,9.45) | 8.49  (7.66,9.40) | 2399.85 | 3.07  (2.89) | 8.41  (7.58) |
| Injury, poisoning and procedural complications | Exposure during pregnancy | 363 | 3.18  (2.87,3.52) | 3.17  (2.86,3.51) | 537.22 | 1.66  (1.50) | 3.16  (2.85) |
| General disorders and administration site conditions | Drug withdrawal syndrome | 350 | 3.25  (2.92,3.61) | 3.24  (2.92,3.60) | 539.93 | 1.69  (1.53) | 3.23  (2.91) |
| Psychiatric disorders | Completed suicide | 348 | 3.51  (3.16,3.90) | 3.50  (3.15,3.89) | 619.77 | 1.80  (1.64) | 3.49  (3.14) |
| Psychiatric disorders | Restlessness | 346 | 7.99  (7.18,8.88) | 7.95  (7.16,8.84) | 2084.20 | 2.98  (2.79) | 7.89  (7.09) |
| Psychiatric disorders | Nervousness | 324 | 5.15  (4.62,5.75) | 5.13  (4.60,5.73) | 1072.63 | 2.35  (2.17) | 5.11  (4.58) |
| Reproductive system and breast disorders | Sexual dysfunction | 303 | 22.63  (20.18,25.37) | 22.54  (20.11,25.26) | 6067.02 | 4.46  (4.19) | 21.95  (19.58) |
| Psychiatric disorders | Aggression | 280 | 4.68  (4.16,5.26) | 4.67  (4.15,5.25) | 802.35 | 2.22  (2.02) | 4.64  (4.13) |
| Gastrointestinal disorders | Dry mouth | 270 | 2.84  (2.52,3.20) | 2.84  (2.52,3.19) | 320.06 | 1.50  (1.31) | 2.83  (2.51) |
| Psychiatric disorders | Abnormal behaviour | 267 | 5.60  (4.96,6.31) | 5.58  (4.95,6.29) | 997.19 | 2.47  (2.27) | 5.55  (4.92) |
| Psychiatric disorders | Loss of libido | 258 | 24.98  (22.07,28.28) | 24.90  (22.00,28.18) | 5740.68 | 4.60  (4.29) | 24.18  (21.36) |
| Nervous system disorders | Serotonin syndrome | 253 | 11.42  (10.09,12.93) | 11.39  (10.06,12.89) | 2364.08 | 3.49  (3.25) | 11.24  (9.93) |
| Nervous system disorders | Amnesia | 248 | 3.18  (2.81,3.61) | 3.18  (2.80,3.60) | 368.81 | 1.66  (1.47) | 3.17  (2.80) |
| Psychiatric disorders | Anger | 248 | 6.12  (5.40,6.93) | 6.10  (5.38,6.91) | 1049.55 | 2.60  (2.39) | 6.06  (5.35) |
| Psychiatric disorders | Bruxism | 234 | 38.16  (33.46,43.52) | 38.04  (33.37,43.36) | 8057.83 | 5.18  (4.79) | 36.36  (31.89) |
| Psychiatric disorders | Decreased interest | 234 | 34.31  (30.10,39.12) | 34.21  (30.02,38.98) | 7235.22 | 5.04  (4.66) | 32.85  (28.81) |
| Reproductive system and breast disorders | Erectile dysfunction | 226 | 7.50  (6.57,8.55) | 7.48  (6.56,8.52) | 1256.46 | 2.89  (2.66) | 7.42  (6.50) |
| Injury, poisoning and procedural complications | Intentional overdose | 223 | 2.84  (2.49,3.24) | 2.84  (2.49,3.24) | 264.93 | 1.50  (1.30) | 2.83  (2.48) |
| Ear and labyrinth disorders | Tinnitus | 220 | 4.04  (3.53,4.61) | 4.03  (3.53,4.60) | 498.45 | 2.00  (1.79) | 4.01  (3.51) |
| Congenital, familial and genetic disorders | Congenital anomaly | 219 | 24.78  (21.65,28.35) | 24.70  (21.60,28.26) | 4832.77 | 4.58  (4.24) | 24.00  (20.97) |
| Psychiatric disorders | Mania | 214 | 11.08  (9.68,12.69) | 11.05  (9.66,12.65) | 1930.71 | 3.45  (3.18) | 10.92  (9.54) |
| Psychiatric disorders | Intentional self-injury | 207 | 8.60  (7.50,9.87) | 8.58  (7.49,9.84) | 1372.53 | 3.09  (2.84) | 8.50  (7.41) |
| Skin and subcutaneous tissue disorders | Night sweats | 206 | 5.51  (4.80,6.32) | 5.50  (4.80,6.31) | 753.47 | 2.45  (2.22) | 5.47  (4.77) |
| Psychiatric disorders | Tension | 205 | 28.93  (25.16,33.26) | 28.85  (25.10,33.16) | 5320.65 | 4.80  (4.42) | 27.88  (24.25) |
| Injury, poisoning and procedural complications | Medication error | 202 | 3.10  (2.70,3.56) | 3.10  (2.70,3.55) | 285.85 | 1.63  (1.41) | 3.09  (2.69) |
| Psychiatric disorders | Anorgasmia | 199 | 66.06  (57.16,76.34) | 65.88  (57.02,76.11) | 11749.2 | 5.93  (5.34) | 60.95  (52.74) |
| General disorders and administration site conditions | Therapeutic response unexpected | 199 | 3.36  (2.92,3.86) | 3.35  (2.92,3.85) | 327.00 | 1.74  (1.52) | 3.34  (2.91) |

**Sup table 5.** Signal strength of Sertraline-associated adverse events at the Preferred Term (PT) level ranked by EBGM

| **System Organ Class(SOC)** | **Preferred Term(PT)** | **Case reports** | **ROR (95% CI)** | **PRR (95% CI)** | **Chi Square** | **IC (IC025)** | **EBGM (EBGM05)** |
| --- | --- | --- | --- | --- | --- | --- | --- |
| Congenital, familial and genetic disorders | Inborn error of lipid metabolism | 11 | 8810.36  (1137.42,68244.3) | 8809.05  (1137.28,68232.4) | 8073.13 | 9.52  (2.41) | 735.00  (94.89) |
| Congenital, familial and genetic disorders | Multiple acyl-coenzyme A dehydrogenase deficiency | 178 | 2976.89  (2164.15,4094.87) | 2969.72  (2159.28,4084.33) | 112196 | 9.30  (6.84) | 631.52  (459.11) |
| Investigations | Antidepressant drug clearance decreased | 11 | 1101.30  (442.96,2738.06) | 1101.13  (442.92,2737.49) | 5090.72 | 8.86  (2.50) | 464.21  (186.71) |
| Neoplasms benign, malignant and unspecified (incl cysts and polyps) | Pineocytoma | 5 | 1001.10  (268.81,3728.19) | 1001.03  (268.81,3727.79) | 2220.07 | 8.80  (1.08) | 445.46  (119.61) |
| Neoplasms benign, malignant and unspecified (incl cysts and polyps) | Testicle adenoma | 5 | 1001.10  (268.81,3728.19) | 1001.03  (268.81,3727.79) | 2220.07 | 8.80  (1.08) | 445.46  (119.61) |
| Reproductive system and breast disorders | Scrotal angiokeratoma | 4 | 800.87  (200.29,3202.35) | 800.82  (200.28,3202.06) | 1597.65 | 8.65  (0.70) | 400.91  (100.26) |
| Gastrointestinal disorders | Collagenous gastritis | 9 | 655.30  (271.54,1581.41) | 655.22  (271.52,1581.13) | 3233.44 | 8.50  (2.18) | 360.82  (149.51) |
| Psychiatric disorders | Chronic tic disorder | 6 | 600.67  (208.41,1731.22) | 600.62  (208.40,1731.00) | 2052.41 | 8.42  (1.46) | 343.64  (119.23) |
| Congenital, familial and genetic disorders | Long-chain acyl-coenzyme A dehydrogenase deficiency | 5 | 572.05  (181.55,1802.48) | 572.02  (181.55,1802.27) | 1662.55 | 8.38  (1.14) | 334.09  (106.03) |
| Vascular disorders | Subgaleal haemorrhage | 4 | 400.43  (120.58,1329.85) | 400.41  (120.57,1329.72) | 1062.44 | 8.06  (0.78) | 267.27  (80.48) |
| Musculoskeletal and connective tissue disorders | Muscle hypoxia | 4 | 355.94  (109.61,1155.86) | 355.92  (109.61,1155.75) | 980.10 | 7.95  (0.79) | 246.71  (75.97) |
| Neoplasms benign, malignant and unspecified (incl cysts and polyps) | Anaplastic large cell lymphoma T- and null-cell types stage IV | 3 | 343.22  (88.75,1327.34) | 343.21  (88.75,1327.23) | 716.55 | 7.91  (0.30) | 240.55  (62.20) |
| Congenital, familial and genetic disorders | Corrected transposition of great vessels | 6 | 320.35  (124.29,825.70) | 320.33  (124.29,825.58) | 1364.28 | 7.84  (1.52) | 229.09  (88.88) |
| Musculoskeletal and connective tissue disorders | Mitochondrial myopathy acquired | 14 | 295.10  (159.89,544.64) | 295.04  (159.87,544.49) | 2998.06 | 7.75  (2.97) | 215.88  (116.97) |
| Reproductive system and breast disorders | Genital dysaesthesia | 5 | 286.03  (103.02,794.13) | 286.01  (103.02,794.04) | 1046.36 | 7.72  (1.21) | 211.01  (76.00) |
| Investigations | Anti-transglutaminase antibody | 3 | 218.41  (60.93,782.93) | 218.41  (60.93,782.87) | 510.11 | 7.42  (0.35) | 171.82  (47.93) |
| Eye disorders | Cycloplegia | 7 | 200.22  (87.46,458.39) | 200.21  (87.46,458.32) | 1109.98 | 7.33  (1.81) | 160.36  (70.05) |
| Nervous system disorders | Subdural haemorrhage neonatal | 4 | 200.22  (66.93,598.90) | 200.21  (66.93,598.84) | 634.27 | 7.33  (0.85) | 160.36  (53.61) |
| Endocrine disorders | Pubertal failure | 3 | 200.21  (56.50,709.51) | 200.21  (56.50,709.46) | 475.71 | 7.33  (0.36) | 160.36  (45.25) |
| Gastrointestinal disorders | Allergic colitis | 9 | 189.69  (91.72,392.30) | 189.67  (91.72,392.23) | 1365.63 | 7.26  (2.23) | 153.54  (74.24) |
| Reproductive system and breast disorders | Genital anaesthesia | 42 | 170.83  (122.42,238.38) | 170.73  (122.37,238.21) | 5841.66 | 7.14  (4.57) | 140.91  (100.98) |
| Surgical and medical procedures | Antiandrogen therapy | 6 | 160.18  (66.67,384.85) | 160.16  (66.67,384.79) | 790.85 | 7.06  (1.55) | 133.64  (55.62) |
| Injury, poisoning and procedural complications | Subarachnoid haemorrhage neonatal | 4 | 139.28  (48.17,402.75) | 139.27  (48.17,402.71) | 467.77 | 6.89  (0.87) | 118.79  (41.08) |
| Pregnancy, puerperium and perinatal conditions | Foetal vascular malperfusion | 18 | 133.50  (81.05,219.90) | 133.47  (81.04,219.82) | 2028.52 | 6.84  (3.33) | 114.55  (69.54) |
| Psychiatric disorders | Feelings of worthlessness | 171 | 123.10  (104.78,144.62) | 122.82  (104.57,144.24) | 17914.1 | 6.74  (5.81) | 106.62  (90.75) |
| Psychiatric disorders | Feeling guilty | 167 | 118.83  (100.99,139.82) | 118.56  (100.79,139.46) | 16957.1 | 6.69  (5.77) | 103.40  (87.88) |
| Reproductive system and breast disorders | Female sexual dysfunction | 23 | 100.13  (64.91,154.48) | 100.10  (64.90,154.41) | 2005.87 | 6.48  (3.63) | 89.09  (57.75) |
| Congenital, familial and genetic disorders | Congenital tricuspid valve stenosis | 3 | 100.11  (30.14,332.46) | 100.10  (30.14,332.43) | 261.63 | 6.48  (0.41) | 89.09  (26.83) |
| Respiratory, thoracic and mediastinal disorders | Neonatal anoxia | 6 | 98.07  (42.01,228.93) | 98.06  (42.01,228.90) | 513.54 | 6.45  (1.55) | 87.47  (37.47) |
| Gastrointestinal disorders | Enteric neuropathy | 3 | 92.41  (27.97,305.31) | 92.40  (27.97,305.28) | 243.18 | 6.37  (0.41) | 82.95  (25.11) |
| Nervous system disorders | Juvenile myoclonic epilepsy | 6 | 87.37  (37.62,202.93) | 87.36  (37.62,202.90) | 461.86 | 6.30  (1.55) | 78.87  (33.96) |
| Congenital, familial and genetic disorders | Newborn persistent pulmonary hypertension | 26 | 85.01  (56.74,127.38) | 84.99  (56.73,127.32) | 1950.90 | 6.27  (3.75) | 76.93  (51.34) |
| Neoplasms benign, malignant and unspecified (incl cysts and polyps) | Intracranial meningioma malignant | 4 | 84.30  (30.09,236.21) | 84.30  (30.09,236.18) | 297.88 | 6.25  (0.88) | 76.36  (27.25) |
| Psychiatric disorders | Female orgasmic disorder | 30 | 82.88  (56.91,120.70) | 82.84  (56.89,120.64) | 2198.28 | 6.23  (3.93) | 75.17  (51.61) |
| Surgical and medical procedures | Local anaesthesia | 5 | 81.72  (32.56,205.11) | 81.72  (32.56,205.08) | 361.73 | 6.21  (1.25) | 74.24  (29.58) |
| Nervous system disorders | Neonatal behavioural syndrome | 26 | 81.68  (54.56,122.29) | 81.65  (54.55,122.23) | 1879.64 | 6.21  (3.74) | 74.19  (49.55) |
| General disorders and administration site conditions | High-pitched crying | 6 | 75.08  (32.52,173.38) | 75.08  (32.52,173.35) | 400.95 | 6.10  (1.54) | 68.73  (29.76) |
| Nervous system disorders | Atypical migraine | 3 | 75.08  (22.99,245.19) | 75.08  (22.99,245.17) | 200.48 | 6.10  (0.42) | 68.73  (21.05) |
| Congenital, familial and genetic disorders | Congenital mitral valve stenosis | 4 | 74.50  (26.74,207.55) | 74.50  (26.74,207.53) | 265.35 | 6.09  (0.88) | 68.24  (24.49) |
| Psychiatric disorders | Loss of dreaming | 11 | 74.04  (39.92,137.31) | 74.03  (39.92,137.28) | 725.38 | 6.08  (2.50) | 67.85  (36.58) |
| Injury, poisoning and procedural complications | Deprescribing error | 5 | 70.25  (28.16,175.26) | 70.25  (28.16,175.24) | 313.78 | 6.01  (1.24) | 64.66  (25.92) |
| Reproductive system and breast disorders | Noninfective oophoritis | 5 | 67.87  (27.24,169.11) | 67.87  (27.24,169.09) | 303.67 | 5.97  (1.24) | 62.64  (25.14) |
| Metabolism and nutrition disorders | Lipid metabolism disorder | 26 | 67.63  (45.31,100.92) | 67.60  (45.31,100.87) | 1573.24 | 5.96  (3.67) | 62.42  (41.83) |
| Psychiatric disorders | Anorgasmia | 199 | 66.06  (57.16,76.34) | 65.88  (57.02,76.11) | 11749.2 | 5.93  (5.34) | 60.95  (52.74) |
| Congenital, familial and genetic disorders | Shone complex | 5 | 64.59  (25.97,160.65) | 64.58  (25.97,160.63) | 289.63 | 5.90  (1.24) | 59.84  (24.06) |
| Reproductive system and breast disorders | Genital hypoaesthesia | 71 | 62.54  (49.12,79.63) | 62.48  (49.08,79.54) | 3984.53 | 5.86  (4.66) | 58.03  (45.58) |
| Respiratory, thoracic and mediastinal disorders | Pulmonary microemboli | 7 | 61.61  (28.56,132.90) | 61.60  (28.56,132.88) | 387.52 | 5.84  (1.77) | 57.27  (26.55) |
| Congenital, familial and genetic disorders | Metabolic myopathy | 3 | 61.60  (19.04,199.35) | 61.60  (19.04,199.34) | 166.08 | 5.84  (0.42) | 57.27  (17.70) |
| Reproductive system and breast disorders | Ejaculation delayed | 38 | 60.29  (43.35,83.84) | 60.26  (43.34,83.79) | 2059.55 | 5.81  (4.06) | 56.11  (40.35) |
| Nervous system disorders | Perinatal stroke | 3 | 60.06  (18.58,194.17) | 60.06  (18.58,194.15) | 162.08 | 5.81  (0.42) | 55.94  (17.31) |

**Sup table 6.** Signal strength of Paroxetine associated adverse events at the Preferred Term (PT) level ranked by number of reports from Vigi base

| **System Organ Class (SOC)** | **Preferred Term (PT)** | **Case reports (N)** | **ROR (95% CI)** | **PRR (95% CI)** | **Chi Square** | **IC (IC025)** | **EBGM (EBGM05)** |
| --- | --- | --- | --- | --- | --- | --- | --- |
| General disorders and administration site conditions | Drug withdrawal syndrome | 8468 | 29.75  (29.09,30.42) | 28.66  (28.05,29.28) | 214648 | 4.77  (4.73) | 27.23  (26.63) |
| Psychiatric disorders | Anxiety | 3788 | 4.60  (4.46,4.75) | 4.54  (4.40,4.69) | 10411.7 | 2.17  (2.12) | 4.51  (4.37) |
| Nervous system disorders | Tremor | 3491 | 4.50  (4.35,4.65) | 4.44  (4.30,4.59) | 9270.06 | 2.14  (2.09) | 4.41  (4.27) |
| Psychiatric disorders | Insomnia | 3081 | 3.33  (3.21,3.45) | 3.30  (3.18,3.41) | 4918.79 | 1.71  (1.66) | 3.28  (3.17) |
| Injury, poisoning and procedural complications | Exposure during pregnancy | 3069 | 13.58  (13.10,14.08) | 13.41  (12.94,13.89) | 34398.0 | 3.71  (3.65) | 13.10  (12.63) |
| Skin and subcutaneous tissue disorders | Hyperhidrosis | 2744 | 3.57  (3.44,3.70) | 3.54  (3.41,3.67) | 4975.05 | 1.82  (1.76) | 3.52  (3.39) |
| Nervous system disorders | Paraesthesia | 2655 | 3.10  (2.98,3.22) | 3.07  (2.96,3.19) | 3706.10 | 1.61  (1.56) | 3.06  (2.95) |
| Psychiatric disorders | Depression | 2474 | 3.98  (3.83,4.15) | 3.95  (3.80,4.11) | 5428.45 | 1.97  (1.91) | 3.93  (3.78) |
| Investigations | Weight increased | 2433 | 4.38  (4.21,4.56) | 4.35  (4.18,4.52) | 6233.10 | 2.11  (2.05) | 4.32  (4.15) |
| Psychiatric disorders | Suicidal ideation | 2377 | 12.83  (12.32,13.37) | 12.71  (12.20,13.23) | 25050.2 | 3.64  (3.57) | 12.43  (11.93) |
| Psychiatric disorders | Agitation | 2256 | 7.23  (6.93,7.53) | 7.16  (6.87,7.47) | 11818.4 | 2.82  (2.76) | 7.08  (6.79) |
| Psychiatric disorders | Confusional state | 2035 | 4.00  (3.83,4.18) | 3.97  (3.80,4.15) | 4497.76 | 1.98  (1.91) | 3.95  (3.78) |
| Injury, poisoning and procedural complications | Fetal exposure during pregnancy | 1962 | 11.91  (11.38,12.46) | 11.81  (11.30,12.35) | 19005.8 | 3.53  (3.46) | 11.57  (11.07) |
| General disorders and administration site conditions | Withdrawal syndrome | 1823 | 15.07  (14.38,15.79) | 14.96  (14.28,15.67) | 23096.3 | 3.86  (3.79) | 14.57  (13.90) |
| Metabolism and nutritional disorders | Hyponatraemia | 1742 | 8.47  (8.08,8.89) | 8.42  (8.03,8.82) | 11213.9 | 3.05  (2.98) | 8.30  (7.91) |
| General disorders and administration site conditions | Feeling abnormal | 1696 | 2.73  (2.61,2.87) | 2.72  (2.59,2.85) | 1840.78 | 1.44  (1.37) | 2.71  (2.58) |
| Psychiatric disorders | Aggression | 1554 | 11.02  (10.48,11.59) | 10.95  (10.41,11.51) | 13770.9 | 3.43  (3.34) | 10.75  (10.22) |
| Psychiatric disorders | Completed suicide | 1368 | 4.81  (4.56,5.07) | 4.79  (4.54,5.05) | 4067.58 | 2.25  (2.17) | 4.75  (4.51) |
| Psychiatric disorders | Suicide attempt | 1292 | 7.64  (7.23,8.07) | 7.60  (7.20,8.03) | 7308.83 | 2.91  (2.82) | 7.51  (7.11) |
| Ear and labyrinth disorders | Vertigo | 1232 | 3.17  (2.99,3.35) | 3.15  (2.98,3.33) | 1803.77 | 1.65  (1.57) | 3.14  (2.97) |
| Congenital, familial and genetic disorders | Congenital anomaly | 1177 | 57.02  (53.68,60.56) | 56.72  (53.42,60.23) | 58155.5 | 5.68  (5.53) | 51.29  (48.29) |
| Psychiatric disorders | Nightmare | 1174 | 10.70  (10.10,11.34) | 10.65  (10.06,11.28) | 10069.0 | 3.39  (3.29) | 10.46  (9.87) |
| Congenital, familial and genetic disorders | Atrial septal defect | 1158 | 92.83  (87.20,98.83) | 92.35  (86.78,98.29) | 89003.3 | 6.30  (6.11) | 78.70  (73.92) |
| Nervous system disorders | Disturbance in attention | 1097 | 5.67  (5.34,6.01) | 5.64  (5.32,5.99) | 4150.42 | 2.48  (2.39) | 5.59  (5.27) |
| Psychiatric disorders | Nervousness | 1090 | 5.27  (4.96,5.59) | 5.25  (4.94,5.57) | 3712.76 | 2.38  (2.29) | 5.20  (4.90) |
| Psychiatric disorders | Irritability | 1081 | 4.18  (3.94,4.44) | 4.17  (3.93,4.43) | 2586.96 | 2.05  (1.96) | 4.14  (3.90) |
| Gastrointestinal disorders | Dry mouth | 1007 | 2.40  (2.26,2.55) | 2.39  (2.25,2.55) | 815.69 | 1.26  (1.16) | 2.39  (2.24) |
| General disorders and administration site conditions | Crying | 945 | 2.93  (2.75,3.13) | 2.93  (2.74,3.12) | 1192.62 | 1.54  (1.45) | 2.91  (2.73) |
| Psychiatric disorders | Hallucination | 919 | 3.69  (3.45,3.93) | 3.67  (3.44,3.92) | 1778.58 | 1.87  (1.77) | 3.66  (3.43) |
| Ear and labyrinth disorders | Tinnitus | 904 | 3.13  (2.93,3.34) | 3.12  (2.92,3.33) | 1295.38 | 1.64  (1.54) | 3.11  (2.91) |
| Psychiatric disorders | Libido decreased | 867 | 18.10  (16.91,19.37) | 18.03  (16.86,19.29) | 13489.6 | 4.13  (4.00) | 17.47  (16.32) |
| Nervous system disorders | Serotonin syndrome | 852 | 20.46  (19.10,21.91) | 20.39  (19.04,21.83) | 15122.6 | 4.30  (4.17) | 19.66  (18.36) |
| General disorders and administration site conditions | Drug interaction | 811 | 2.52  (2.36,2.70) | 2.52  (2.35,2.70) | 740.21 | 1.33  (1.22) | 2.51  (2.34) |
| Psychiatric disorders | Sleep disorder | 809 | 2.70  (2.52,2.90) | 2.70  (2.52,2.89) | 859.44 | 1.43  (1.32) | 2.69  (2.51) |
| Psychiatric disorders | Abnormal dreams | 801 | 10.34  (9.64,11.09) | 10.31  (9.61,11.05) | 6604.26 | 3.34  (3.22) | 10.13  (9.44) |
| Psychiatric disorders | Anger | 794 | 10.58  (9.87,11.36) | 10.55  (9.84,11.32) | 6731.79 | 3.37  (3.25) | 10.36  (9.66) |
| Congenital, familial and genetic disorders | Ventricular septal defect | 772 | 87.07  (80.67,93.97) | 86.77  (80.42,93.62) | 56175.5 | 6.22  (5.98) | 74.61  (69.13) |
| Psychiatric disorders | Panic attack | 761 | 8.57  (7.97,9.20) | 8.54  (7.95,9.17) | 4987.54 | 3.07  (2.95) | 8.42  (7.84) |
| Nervous system disorders | Memory impairment | 753 | 2.38  (2.22,2.56) | 2.38  (2.21,2.55) | 599.04 | 1.25  (1.14) | 2.37  (2.21) |
| Reproductive system and breast disorders | Sexual dysfunction | 668 | 26.51  (24.53,28.66) | 26.44  (24.46,28.57) | 15566.5 | 4.66  (4.49) | 25.22  (23.33) |
| Nervous system disorders | Amnesia | 659 | 3.42  (3.17,3.70) | 3.42  (3.16,3.69) | 1119.69 | 1.77  (1.65) | 3.40  (3.15) |
| Investigations | Cardiac murmur | 657 | 33.21  (30.68,35.93) | 33.11  (30.60,35.82) | 19248.4 | 4.96  (4.78) | 31.21  (28.84) |
| Nervous system disorders | Dyskinesia | 650 | 4.28  (3.96,4.62) | 4.27  (3.95,4.61) | 1613.00 | 2.08  (1.96) | 4.24  (3.92) |
| Congenital, familial and genetic disorders | Patent ductus arteriosus | 635 | 111.77  (102.59,121.78) | 111.46  (102.32,121.41) | 57346.0 | 6.53  (6.21) | 92.12  (84.55) |
| General disorders and administration site conditions | Adverse event | 629 | 3.38  (3.13,3.66) | 3.38  (3.12,3.65) | 1046.77 | 1.75  (1.63) | 3.36  (3.11) |
| Nervous system disorders | Sedation | 627 | 6.25  (5.78,6.77) | 6.24  (5.77,6.75) | 2726.77 | 2.63  (2.50) | 6.18  (5.71) |
| Reproductive system and breast disorders | Erectile dysfunction | 611 | 6.49  (6.00,7.03) | 6.48  (5.98,7.02) | 2797.81 | 2.68  (2.55) | 6.41  (5.92) |
| Psychiatric disorders | Mania | 563 | 13.53  (12.44,14.71) | 13.50  (12.42,14.68) | 6353.67 | 3.72  (3.57) | 13.19  (12.13) |
| Cardiac disorders | Left atrial dilatation | 15 | 3.58  (2.16,5.95) | 3.58  (2.16,5.95) | 27.75 | 1.83  (0.90) | 3.57  (2.15) |
| Congenital, familial and genetic disorders | Congenital genitourinary abnormality | 15 | 22.98  (13.70,38.53) | 22.97  (13.70,38.53) | 302.05 | 4.46  (2.51) | 22.05  (13.15) |

**Supp table 7.** Signal strength of Paroxetine associated adverse events at the Preferred Term (PT) level ranked by EBGM from Vigi base

| **System Organ Class (SOC)** | **Preferred Term (PT)** | **Case reports (N)** | **ROR (95% CI)** | **PRR (95% CI)** | **Chi Square** | **IC (IC025)** | **EBGM (EBGM05)** |
| --- | --- | --- | --- | --- | --- | --- | --- |
| Congenital, familial and genetic disorders | Congenital aortic atresia | 19 | 475.35  (255.56,884.16) | 475.31  (255.54,884.06) | 4721.24 | 7.97  (3.43) | 250.01  (134.41) |
| Congenital, familial and genetic disorders | Lipomeningocele | 6 | 450.30  (151.33,1339.92) | 450.29  (151.33,1339.87) | 1448.33 | 7.92  (1.43) | 242.92  (81.64) |
| Congenital, familial and genetic disorders | Persistent fetal circulation | 179 | 402.18  (331.04,488.61) | 401.86  (330.80,488.18) | 40553.8 | 7.83  (6.40) | 228.12  (187.77) |
| Congenital, familial and genetic disorders | Congenital Eustachian tube anomaly | 4 | 350.23  (98.83,1241.13) | 350.22  (98.83,1241.09) | 835.75 | 7.72  (0.74) | 210.53  (59.41) |
| Congenital, familial and genetic disorders | Congenital aortic stenosis | 73 | 339.49  (252.93,455.68) | 339.38  (252.86,455.50) | 14962.7 | 7.69  (5.37) | 206.57  (153.90) |
| Congenital, familial and genetic disorders | Congenital mitral valve stenosis | 16 | 254.73  (140.20,462.80) | 254.71  (140.20,462.75) | 2723.11 | 7.43  (3.15) | 171.87  (94.60) |
| Congenital, familial and genetic disorders | Congenital aortic valve incompetence | 34 | 229.03  (153.09,342.63) | 228.99  (153.07,342.56) | 5374.98 | 7.32  (4.29) | 159.78  (106.80) |
| Congenital, familial and genetic disorders | Congenital epiglottal anomaly | 3 | 225.15  (58.22,870.69) | 225.14  (58.22,870.67) | 468.61 | 7.30  (0.29) | 157.90  (40.83) |
| Congenital, familial and genetic disorders | Acrocephalosyndactyly | 3 | 197.00  (52.26,742.60) | 197.00  (52.26,742.58) | 425.47 | 7.17  (0.30) | 143.55  (38.08) |
| Congenital, familial and genetic disorders | Bicuspid aortic valve | 133 | 174.78  (143.64,212.67) | 174.67  (143.56,212.53) | 17235.7 | 7.04  (5.78) | 131.34  (107.94) |
| Congenital, familial and genetic disorders | Pulmonary artery stenosis congenital | 177 | 166.47  (140.57,197.14) | 166.34  (140.48,196.97) | 22093.9 | 6.98  (5.97) | 126.58  (106.89) |
| Congenital, familial and genetic disorders | Cor triatriatum | 4 | 161.65  (52.71,495.76) | 161.64  (52.71,495.74) | 488.34 | 6.95  (0.81) | 123.84  (40.38) |
| Congenital, familial and genetic disorders | Right ventricle outflow tract obstruction | 19 | 158.45  (94.86,264.66) | 158.44  (94.86,264.63) | 2283.66 | 6.93  (3.39) | 121.96  (73.01) |
| Cardiac disorders | Pulmonary valve stenosis | 189 | 155.51  (132.21,182.92) | 155.38  (132.11,182.75) | 22373.1 | 6.91  (5.97) | 120.14  (102.14) |
| Congenital, familial and genetic disorders | Pulmonary valve stenosis congenital | 78 | 155.27  (120.60,199.89) | 155.21  (120.57,199.81) | 9225.47 | 6.91  (5.22) | 120.04  (93.24) |
| Congenital, familial and genetic disorders | Congenital aortic valve stenosis | 23 | 145.59  (91.74,231.06) | 145.58  (91.73,231.03) | 2585.84 | 6.84  (3.67) | 114.21  (71.96) |
| Congenital, familial and genetic disorders | Congenital pulmonary hypertension | 29 | 142.40  (94.47,214.65) | 142.38  (94.46,214.61) | 3203.12 | 6.81  (3.99) | 112.23  (74.46) |
| Cardiac disorders | Sub valvular aortic stenosis | 6 | 131.34  (53.69,321.31) | 131.33  (53.69,321.29) | 620.84 | 6.72  (1.52) | 105.27  (43.03) |
| Congenital, familial and genetic disorders | Aorticopulmonary septal defect | 4 | 131.34  (43.91,392.86) | 131.33  (43.91,392.84) | 413.89 | 6.72  (0.83) | 105.27  (35.19) |
| Congenital, familial and genetic disorders | Coarctation of the aorta | 173 | 120.79  (102.38,142.50) | 120.70  (102.32,142.38) | 16699.2 | 6.62  (5.74) | 98.33  (83.35) |
| Congenital, familial and genetic disorders | Congenital tracheomalacia | 12 | 118.95  (63.57,222.59) | 118.94  (63.57,222.57) | 1144.34 | 6.60  (2.65) | 97.17  (51.93) |
| Congenital, familial and genetic disorders | Hypoplastic left heart syndrome | 107 | 114.30  (92.74,140.89) | 114.25  (92.70,140.81) | 9866.09 | 6.55  (5.36) | 94.02  (76.28) |
| Congenital, familial and genetic disorders | Fallot's tetralogy | 158 | 114.25  (96.19,135.71) | 114.17  (96.13,135.60) | 14560.3 | 6.55  (5.64) | 93.97  (79.11) |
| Congenital, familial and genetic disorders | Abdominal transposition | 5 | 114.21  (43.42,300.41) | 114.20  (43.42,300.40) | 460.87 | 6.55  (1.21) | 93.99  (35.73) |
| Congenital, familial and genetic disorders | Transposition of the great vessels | 132 | 113.37  (93.93,136.84) | 113.31  (93.88,136.75) | 12086.9 | 6.55  (5.51) | 93.38  (77.37) |
| Congenital, familial and genetic disorders | Patent ductus arteriosus | 635 | 111.77  (102.59,121.78) | 111.46  (102.32,121.41) | 57346.0 | 6.53  (6.21) | 92.12  (84.55) |
| Congenital, familial and genetic disorders | Congenital scoliosis | 24 | 109.65  (70.63,170.23) | 109.64  (70.62,170.20) | 2137.41 | 6.51  (3.68) | 90.88  (58.54) |
| Congenital, familial and genetic disorders | Interruption of aortic arch | 12 | 106.85  (57.44,198.77) | 106.85  (57.44,198.76) | 1045.62 | 6.48  (2.65) | 88.96  (47.82) |
| Congenital, familial and genetic disorders | Congenital mitral valve incompetence | 18 | 105.08  (63.34,174.30) | 105.07  (63.34,174.28) | 1546.16 | 6.45  (3.26) | 87.72  (52.88) |
| Congenital, familial and genetic disorders | Velo-cardio-facial syndrome | 4 | 105.07  (35.91,307.40) | 105.07  (35.91,307.39) | 343.59 | 6.45  (0.84) | 87.72  (29.98) |
| Congenital, familial and genetic disorders | Bicuspid pulmonary valve | 3 | 105.07  (30.42,362.94) | 105.07  (30.42,362.93) | 257.69 | 6.45  (0.36) | 87.72  (25.40) |
| Congenital, familial and genetic disorders | Scaphocephaly | 9 | 102.79  (50.31,209.99) | 102.78  (50.31,209.98) | 758.70 | 6.43  (2.19) | 86.13  (42.16) |
| Congenital, familial and genetic disorders | Congenital aortic anomaly | 35 | 101.60  (70.75,145.91) | 101.58  (70.74,145.88) | 2920.98 | 6.41  (4.15) | 85.29  (59.39) |
| Congenital, familial and genetic disorders | Right aortic arch | 24 | 100.88  (65.17,156.13) | 100.86  (65.17,156.11) | 1990.77 | 6.41  (3.66) | 84.78  (54.77) |
| Congenital, familial and genetic disorders | Congenital heart valve disorder | 38 | 100.33  (70.91,141.95) | 100.32  (70.91,141.92) | 3137.30 | 6.40  (4.25) | 84.39  (59.65) |
| Cardiac disorders | Supravalvular aortic stenosis | 4 | 100.07  (34.35,291.52) | 100.06  (34.35,291.51) | 329.53 | 6.40  (0.84) | 84.21  (28.91) |
| Congenital, familial and genetic disorders | Hypoplastic right heart syndrome | 24 | 99.29  (64.18,153.59) | 99.28  (64.18,153.57) | 1963.77 | 6.39  (3.66) | 83.66  (54.08) |
| Congenital, familial and genetic disorders | Ventricular hypoplasia | 32 | 96.63  (66.28,140.88) | 96.61  (66.27,140.85) | 2557.61 | 6.35  (4.03) | 81.76  (56.08) |
| Congenital, familial and genetic disorders | Congenital pulmonary valve disorder | 14 | 95.52  (54.05,168.82) | 95.52  (54.05,168.80) | 1107.93 | 6.34  (2.87) | 80.97  (45.82) |
| Congenital, familial and genetic disorders | Congenital floppy infant | 4 | 95.52  (32.92,277.19) | 95.52  (32.91,277.18) | 316.55 | 6.34  (0.84) | 80.97  (27.90) |
| Congenital, familial and genetic disorders | Congenital tricuspid valve atresia | 23 | 95.15  (61.02,148.36) | 95.14  (61.02,148.34) | 1813.95 | 6.33  (3.59) | 80.71  (51.76) |
| Congenital, familial and genetic disorders | Congenital coronary artery malformation | 22 | 94.74  (60.17,149.18) | 94.73  (60.17,149.16) | 1728.64 | 6.33  (3.52) | 80.41  (51.07) |
| Congenital, familial and genetic disorders | Pilonidal cyst congenital | 9 | 94.56  (46.51,192.29) | 94.56  (46.50,192.28) | 706.05 | 6.33  (2.18) | 80.29  (39.48) |
| Congenital, familial and genetic disorders | Anomalous pulmonary venous connection | 39 | 93.14  (66.26,130.94) | 93.13  (66.25,130.91) | 3019.20 | 6.31  (4.25) | 79.26  (56.38) |
| Congenital, familial and genetic disorders | Atrial septal defect | 1158 | 92.83  (87.20,98.83) | 92.35  (86.78,98.29) | 89003.3 | 6.30  (6.11) | 78.70  (73.92) |
| Injury, poisoning and procedural complications | Electric shock | 144 | 92.77  (77.70,110.75) | 92.71  (77.66,110.67) | 11103.9 | 6.30  (5.42) | 78.95  (66.13) |
| Social circumstances | Promiscuity | 6 | 92.71  (38.92,220.83) | 92.71  (38.92,220.82) | 462.66 | 6.30  (1.52) | 78.95  (33.15) |
| Congenital, familial and genetic disorders | Congenital aortic dilatation | 3 | 92.71  (27.17,316.35) | 92.71  (27.17,316.34) | 231.33 | 6.30  (0.37) | 78.95  (23.14) |
| Congenital, familial and genetic disorders | Double outlet right ventricle | 42 | 92.34  (66.52,128.17) | 92.32  (66.51,128.14) | 3226.80 | 6.30  (4.33) | 78.67  (56.67) |
| Congenital, familial and genetic disorders | Mitral valve dysplasia | 6 | 87.56  (36.89,207.80) | 87.56  (36.89,207.79) | 440.06 | 6.23  (1.52) | 75.19  (31.68) |
